# Supplementary material for: Crossing the blood-brain-barrier with nanoligand drug carriers self-assembled from a phage display peptide
Source: Nat Commun. 2019 Oct 11;10:4635. doi: 10.1038/s41467-019-12554-2 (PMC6789111; doi:10.1038/s41467-019-12554-2)
Supplement: Supplementary file 1 — Supplementary Information [file 41467_2019_12554_MOESM1_ESM.pdf]

Supplementary Information

Crossing the blood-brain-barrier with nanoligand drug carriers self-assembled from a phage display peptide

Wu L. et al.

Supplementary Table 1. Bioenergetic parameters in hCMEC/D3 cells following different treatment strategies

Treatments	Basal respiration	Proton leak	MRR	ATP turnover	Coupling efficiency of OXPHOS	RCR
Control (8h)	187.7 ± 13.5	39.3 ± 9.5	289.1 ± 8.7	148.4 ± 13.3	0.79 ± 0.05	7.7 ± 1.6
2 µM FAM-CGY (8h)	190.6 ± 6.6	42.8 ± 6.0	308.3 ± 21.8	147.7 ± 8.2	0.76 ± 0.03	7.4 ± 1.6
5 µM FAM-CGY (8h)	180.6 ± 13.0	40.5 ± 10.7	294.4 ± 14.0	140.1 ± 18.6	0.77 ± 0.06	7.8 ± 2.3
10 µM FAM-CGY (8h)	190.1 ± 7.9	46.3 ± 14.1	284.4 ± 14.1	143.9 ± 21.2	0.75 ± 0.08	6.8 ± 2.4
20 µM FAM-CGY (8h)	190.1 ± 8.4	36.4 ± 9.2	268.9 ± 12.2	153.8 ± 11.8	0.81 ± 0.05	7.9 ± 2.4
siPORT 1.4 µM (8h)	179.1 ± 8.3	35.9 ± 6.9	269.1 ± 18.6	143.2 ± 12.3	0.80 ± 0.04	7.7 ± 1.5
siPORT 2.8 µM (8h)	157.4 ± 16.6 **	32.8 ± 6.2	275.8 ± 21.2	124.7 ± 14.6	0.79 ± 0.03	8.6 ± 1.2
Control (24h)	188.0 ± 12.1	32.9 ± 8.3	309.2 ± 12.7	155.1 ± 5.8	0.83 ± 0.04	10.0 ± 2.8
2 µM FAM-CGY (24h)	190.6 ± 12.3	34.5 ± 6.3	316.6 ± 23.0	156.1 ± 14.3	0.82 ± 0.03	9.4 ± 1.9
5 µM FAM-CGY (24h)	198.4 ± 22.1	37.9 ± 6.7	310.5 ± 22.3	160.5 ± 23.0	0.81 ± 0.04	8.4 ± 1.6
10 µM FAM-CGY (24h)	204.4 ± 5.5	35.0 ± 6.5	308.3 ± 27.5	169.5 ± 9.7	0.83 ± 0.03	9.0 ± 1.4
20 µM FAM-CGY (24h)	205.8 ± 12.0	36.3 ± 8.8	291.2 ± 19.9	169.6 ± 11.7	0.82 ± 0.04	8.5 ± 2.6
siPORT 1.4 µM (24h)	154.0 ± 13.2 *	34.1 ± 10.9	246.8 ± 20.1 **	120.0 ± 17.2 *	0.78 ± 0.04	7.9 ± 2.5
siPORT 2.8 µM (24h)	111.0 ± 18.1 **	40.0 ± 8.2	157.4 ± 22.3 **	71.0 ± 23.6 **	0.62 ± 0.15 **	4.1 ± 1.2 **
Control + 24 nM siRNA (24h)	195.4 ± 12.8	33.1 ± 6.7	304.5 ± 20.2	162.3 ± 16.4	0.83 ± 0.04	9.5 ± 2.1
FAM-CGY + 8 nM siRNA (24h)	195.6 ± 6.8	36.4 ± 7.7	292.8 ± 18.3	159.2 ± 9.0	0.81 ± 0.04	8.5 ± 2.5
FAM-CGY + 16 nM siRNA (24h)	192.2 ± 9.4	35.9 ± 6.9	301.8 ± 17.1	156.3 ± 11.0	0.81 ± 0.04	8.7 ± 1.8
FAM-CGY + 24 nM siRNA (24h)	181.0 ± 18.2	35.7 ± 8.9	297.5 ± 23.9	145.3 ± 14.4	0.80 ± 0.04	8.8 ± 2.5
siPORT 1.4 µM + 24 nM siRNA (24h)	166.6 ± 9.6 **	37.3 ± 8.4	241.5 ± 22.4 **	129.3 ± 14.2 **	0.77 ± 0.06	6.7 ± 1.2 *
siPORT 2.8 µM + 24 nM siRNA (24h)	89.5 ± 20.8 **	37.1 ± 5.5	150.4 ± 22.2 **	52.4 ± 18.3 **	0.57 ± 0.08 **	4.1 ± 0.8 **

The units for the respiratory states [basal respiration, proton leak, maximum respiratory rate (MRR) and the ATP turnover] are pmol oxygen consumed.min^{-1} per 20,000 cells. The coupling efficiency of OXPHOS and the respiratory control ratio (RCR) are calculated based on the respiratory states as described in Methodology. The results represent mean ± s.d. (triplicate samples) from a typical experiment. The experiment was repeated twice with identical results. Statistical analyses were performed with one-way ANOVA, using Tukey's multiple comparison correction to calculate significance (*$p < 0.05$; **$p < 0.01$; relative to corresponding control).

Supplementary Table 2. Blood count following intravenous injection of FAM-CGY NLCs in free form and complexed with siRNA

Samples	WBC ($\times10^9$)L^{-1}	RBC ($\times10^{12}$)L^{-1}	HGB (gL^{-1})	HCT (%)	MCV (fL)	MCH (pg)	MCHC (gL^{-1})	PLT ($\times10^9$)L^{-1}
Control	4.47±0.54	9.57±0.83	171.00±11.05	77.98±8.78	81.68±2.28	17.93±0.34	219.25±07.25	764.25±57.13
siRNA 4 h	4.84±0.65	9.87±0.14	180.33±4.89	81.23±2.89	82.33±1.98	18.30±0.27	222.67±5.78	718.00±64.67
siRNA 24 h	4.29±0.45	9.52±1.09	177.00±10.00	71.77±08.04	84.63±2.64	18.43±0.69	217.67±6.22	759.33±41.11
siRNA 72 h	4.54±0.30	9.67±0.52	175.33±5.11	70.90±1.60	82.03±3.71	17.97±0.62	219.00±2.00	792.33±63.78
FAM-CGY 4 h	4.44±0.44	9.53±0.78	172.33±10.44	76.83±4.22	80.77±2.24	18.10±0.33	224.33±1.78	766.00±68.00
FAM-CGY 24 h	4.41±0.26	9.78±0.28	170.33±6.22	72.83±2.82	82.97±3.58	18.27±0.84	220.33±1.99	753.00±58.00
FAM-CGY 72 h	4.32±0.53	10.8±0.20	171.67±7.78	75.03±3.56	83.20±2.33	17.97±0.36	216.00±4.00	745.37±56.18
siRNA/FAM-CGY 4 h	4.88±0.42	9.41±0.53	173.67±6.88	78.67±4.84	83.67±1.96	18.53±0.49	221.00±4.23	801.67±54.89
siRNA/FAM-CGY 24 h	4.61±0.52	9.56±0.22	179.00±2.00	71.47±0.64	83.53±0.96	18.60±0.27	223.00±2.67	774.00±60.00
siRNA/FAM-CGY 72 h	4.57±0.64	9.96±0.49	172.67±4.89	79.67±2.49	80.10±2.73	17.37±0.49	216.33±1.78	724.67±48.22
LPS 24 h	1.49±0.26*	8.72±0.61	152.25±14.25	71.90±6.10	82.78±1.23	17.45±0.45	211.75±2.63	282.45±28.83*

WBC: white blood cell; RBC: red blood cell count; HGB: haemoglobin; HCT: haematocrit; MCV: mean corpuscular volume; MCH: mean corpuscular haemoglobin; MCHC: mean corpuscular haemoglobin concentration; PLT: platelet; LPS: lipopolysaccharide.* Statistical analyses were performed with one-way ANOVA, using Tukey's multiple comparison correction to calculate significance (*$p < 0.05$). The results are mean ± s.d. (n = 3 animals per group). The experiment was repeated twice with identical results. The siRNA represent BACE-1-specific siRNA. The final concentration of the peptide conjugate was 10 µM and that of siRNA was 48 nM, based on blood volume calculation equivalent to 6% of the body weight. LPS = lipopolysaccharide.

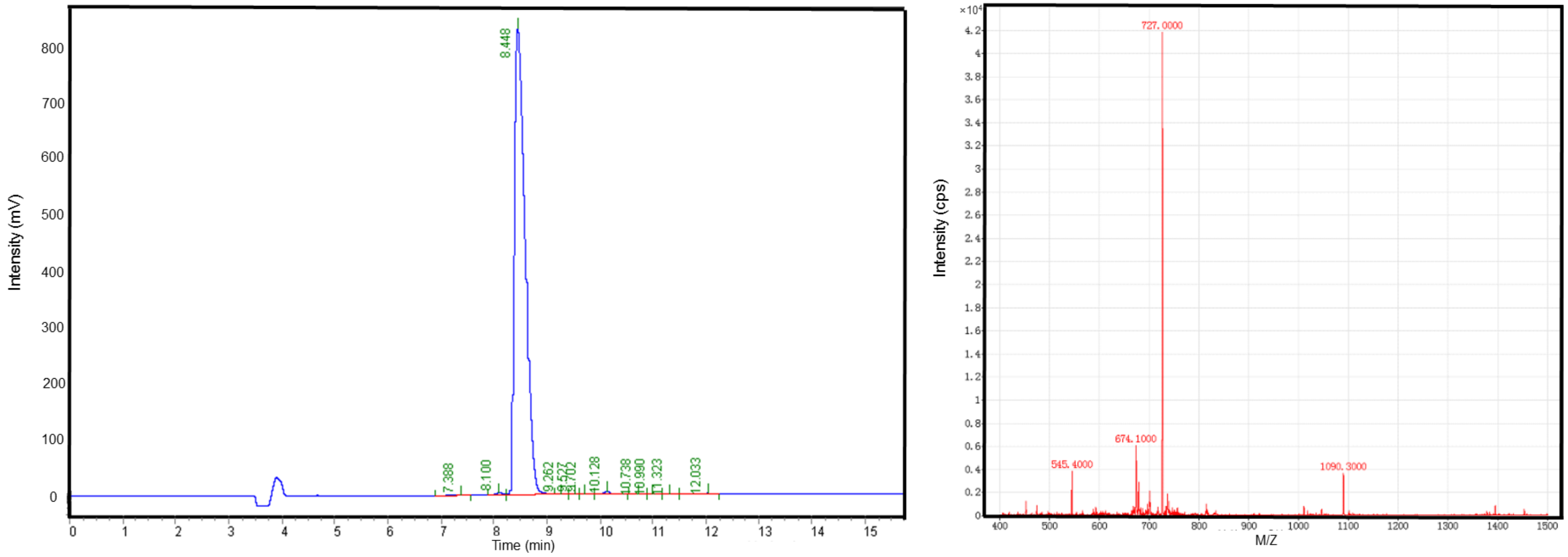

Supplementary Fig. 1. Representative analytical HPLC chromatogram (left) and mass spectra (right) of FAM-CGY conjugate. The peptide was synthesized by a solid-phase method using a Tetras Peptide Synthesizer (CreoSalus, USA). Briefly, for *N*-terminal labelling with 5-carboxy-fluorescein (5-FAM), 2-chlorotrityl chloride resin (Cl-Resin, Genscript) was reacted with three equivalents of 9-fluorenylmethoxycarbonyl (Fmoc)-L-Gly-OH through addition of ten equivalents of N,N-diisopropylethylamine (DIEA, Sigma-Aldrich). Methanol was used to cap the resin and dimethylformamide (DMF, Sigma-Aldrich) to wash the extra amino acid. The Fmoc group was removed by gentle agitation in 20% (v/v) of piperidine in DMF to expose amino group. Three equivalents of Fmoc-L-Pro-OH were coupled to the $–NH_2$ of L-Gly to form amido linkage with three equivalents of N,N,N´,N´-tetramethyl-O-(1H-benzotriazol-1-yl)uronium hexafluorophosphate, O-(benzotriazol-1-yl)-N,N,N´,N´-tetramethyluronium hexafluorophosphate (HBTU, Sigma-Aldrich) and ten equivalents of DIEA in DMF. The peptide synthesise process were repeated until the last amino acid was conjugated. Five equivalents of 5-FAM in DMF were coupled to the *N*-terminal NH_2 group of the peptide with light protection for 4 h by adding three equivalents of HBTU and ten equivalents of DIEA. After 5-FAM conjugation,

cleavage of the peptides from the resin was carried out with a mixture of trifluoroacetic acid (TFA, Merck), triisopropylsilane (Tri, Merck), 1,2-ethanedithiol (EDT, Merck) and deionized water in a volume ratio of 95:1:2:2 for 2 h. The solution was concentrated by rotary evaporation, followed by precipitation in cold diethyl ether (Sigma-Aldrich). The crude peptide was collected by filtration, dried under vacuum and further purified using preparative high-performance liquid chromatography (HPLC) (Shimadzu LC-8A) equipped with a C18 column (Grace™ Vydac™, 10×250 mm). The mobile phase was composed of HPLC grade water containing 0.1% (v/v) TFA and acetonitrile containing 0.1% (v/v) TFA and the volume percentage of acetonitrile was gradually increased from 5 to 95% at 45 min, at a flow rate of 10 $mLmin^{-1}$. (a) Shows a representative analytical HPLC chromatogram of FAM-CGY. (b) Is a typical representative mass spectrum of FAM-CGY. The peaks in the mass spectrum represent charged particles with mass-to-charge ratios of $(M+4H)^{4+}$ (545.4000), $(M+3H)^{3+}$ (727.0000) and $(M+2H)^{2+}$ (1090.3000). Repeated three times with different preparations of FAM-CGY showing identical results.

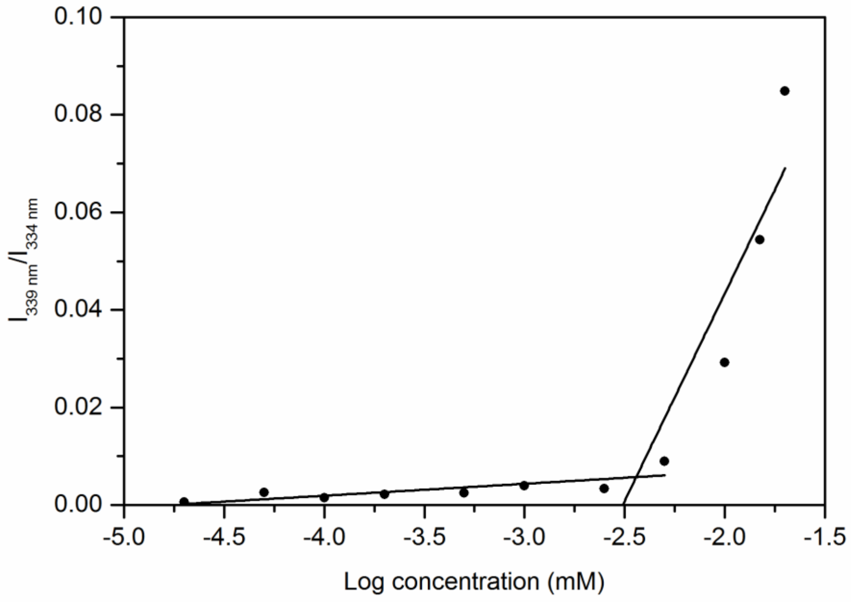

Supplementary Fig. 2. The critical aggregation concentration (CAC) of FAM-CGY. CAC was determined by fluorescence spectroscopy using pyrene (n = 3 separate determinations, duplicate samples). The intersection of two best-fit straight lines represent the CAC. Repeated three times each with a new batch of FAM-CGY and identical spectra were obtained.

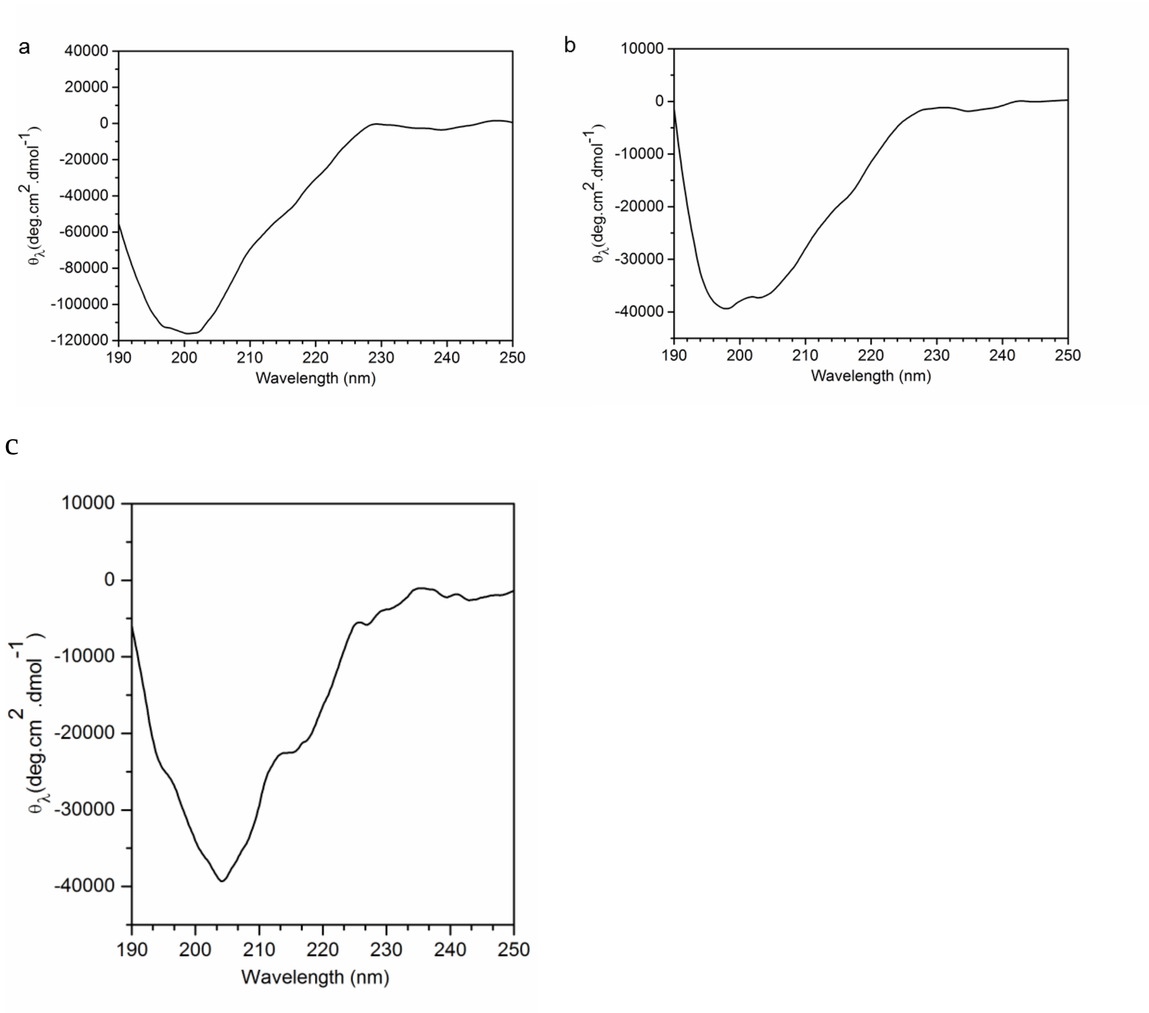

Supplementary Fig. 3. Circular dichroism (CD) spectra of CGY and FAM-CGY. The CD spectra relates to 10 μM CGY (a), 2 μM FAM-CGY (b) and 10 μM FAM-CGY (c) and measured with Jasco J-810 Spectropolarimeter (Jacso Incorporated, MD, USA) with 4 s accumulations every 1 nm and averaged over 3 acquisitions. Rrepeated three times each with a new batch of peptide and with identical results.

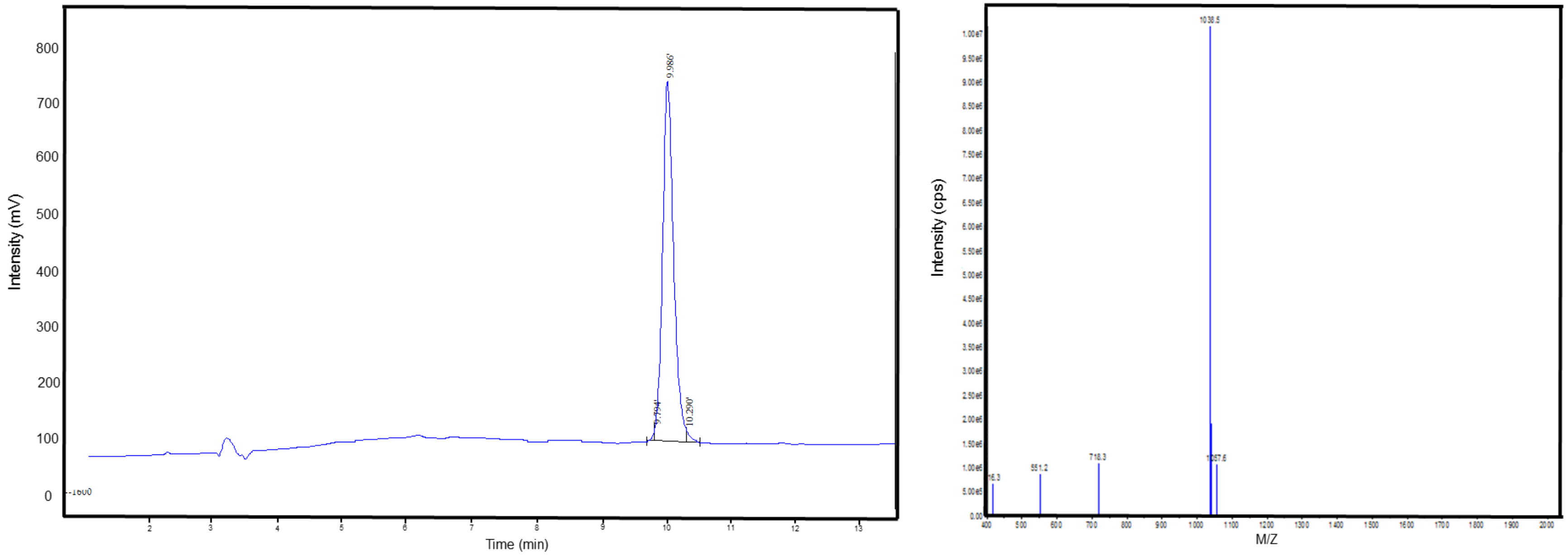

Supplementary Fig. 4. Representative analytical HPLC chromatogram (left) and mass spectra (right) of FAM-GYR peptide. Experimental details were the same as in Supplementary Fig. 1. Panel (a) shows a representative analytical HPLC chromatogram of FAM-GYR. Panel (b) is a typical representative mass spectrum of FAM-GYR. The peak in the mass spectrum represent charged particles with mass-to-charge ratios $(M+2H)^{2+}$ (1038.5). Repeated twice with different preparations of FAM-GYR with identical results.

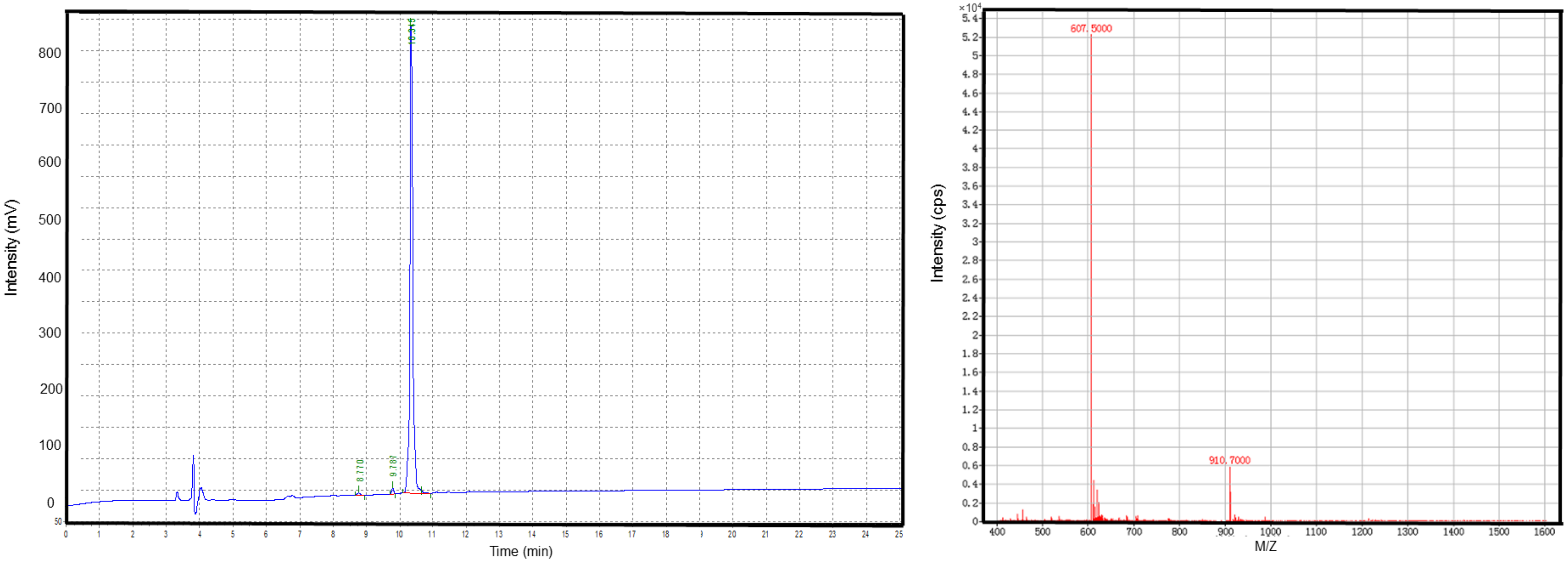

Supplementary Fig. 5. Representative analytical HPLC chromatogram (left) and mass spectra (right) of the CGY peptide. Experimental details were the same as in Supplementary Fig. 1. Panel (a) shows a representative analytical HPLC chromatogram of CGY. Panel (b) is a typical representative mass spectrum of CGY. The peaks in the mass spectrum represent charged particles with mass-to-charge ratios of $(M+3H)^{3+}$ (607.5000) and $(M+2H)^{2+}$ (910.7000). Repeated twice with different preparations of CGY with identical results.

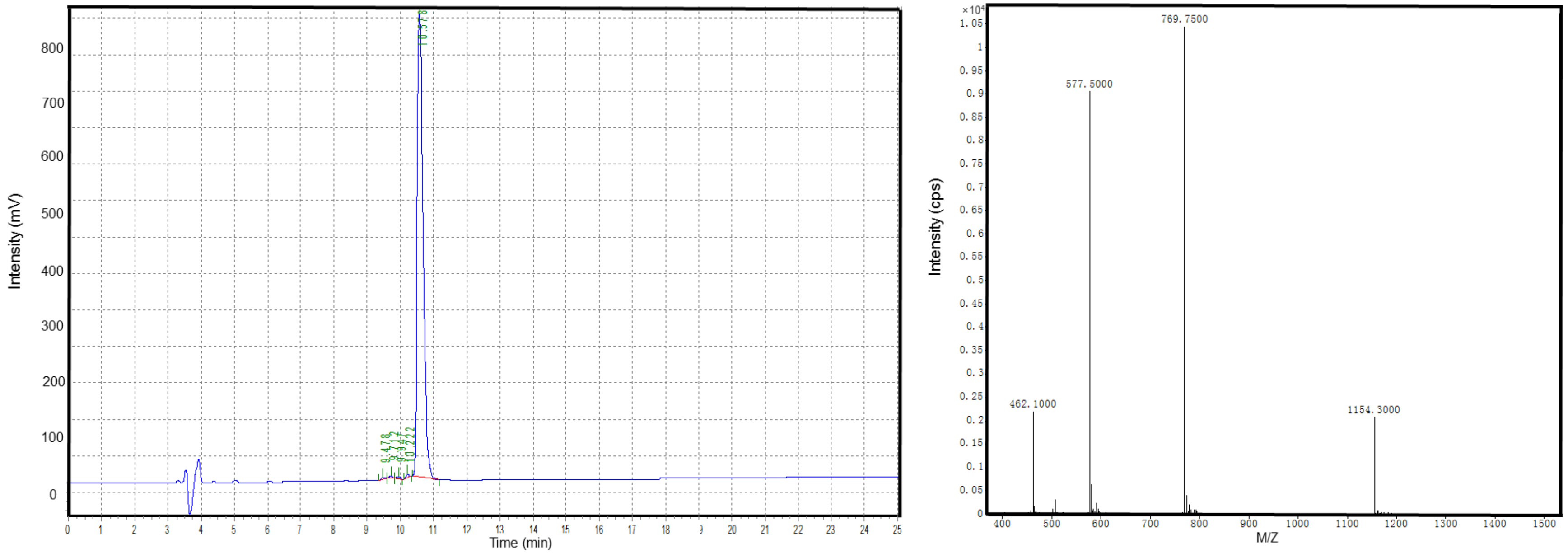

Supplementary Fig. 6. Representative analytical HPLC chromatogram (left) and mass spectra (right) of CGY-(K)-FAM peptide. For *C*-terminal labelling with 5-FAM, Cl-Resin was reacted with three equivalents of Fmoc-L-Lys(Dde)-OH by adding ten equivalents of DIEA in DMF. Methanol was used to cap the resin. The Fmoc group was removed by gentle agitation in 20% (v/v) of piperidine in DMF to expose –NH_2. The three equivalents of Fmoc-L-Gly-OH were coupled to the –NH_2 of L-Lys to form amido linkage with three equivalents of HBTU and ten equivalents of DIEA in DMF. The overall peptide synthesis process was the same as in Supplementary Fig. 1 until last amino acid (Cys) was coupled. Afterward, three equivalents of di-tert-butyl dicarbonate (Boc_2O, Sigma-Aldrich) were reacted with -NH_2 group of Cys for 30 min to form Boc protection of amines under ten equivalents of DIEA in DMF. Next, remaining Boc_2O were washed twice by DMF and methanol. 2% (v/v) hydrazine hydrate (Sigma-Aldrich) in DMF were used to orthogonally de-protect Dde group in lysine (Fmoc-Lys(Dde)-OH). Five equivalents of 5-FAM were then reacted with the –NH_2 group in side chain of Lys under three equivalents of HBTU and ten equivalents of DIEA in DMF for 30 min with light protection. Panel (a) shows a representative analytical

HPLC chromatogram of CGY-(K)-FAM. Panel (b) is a typical representative mass spectra of CGY-(K)-FAM. The peaks in the mass spectrum represent charged particles with mass-to-charge ratios of $(M+5H)^{5+}$ (462.1000), $(M+4H)^{4+}$ (577.5000), $(M+3H)^{3+}$ (769.7500) and $(M+2H)^{2+}$ (1154.3000). Repeated twice with different preparations of CGY-(K)-FAM with identical results.

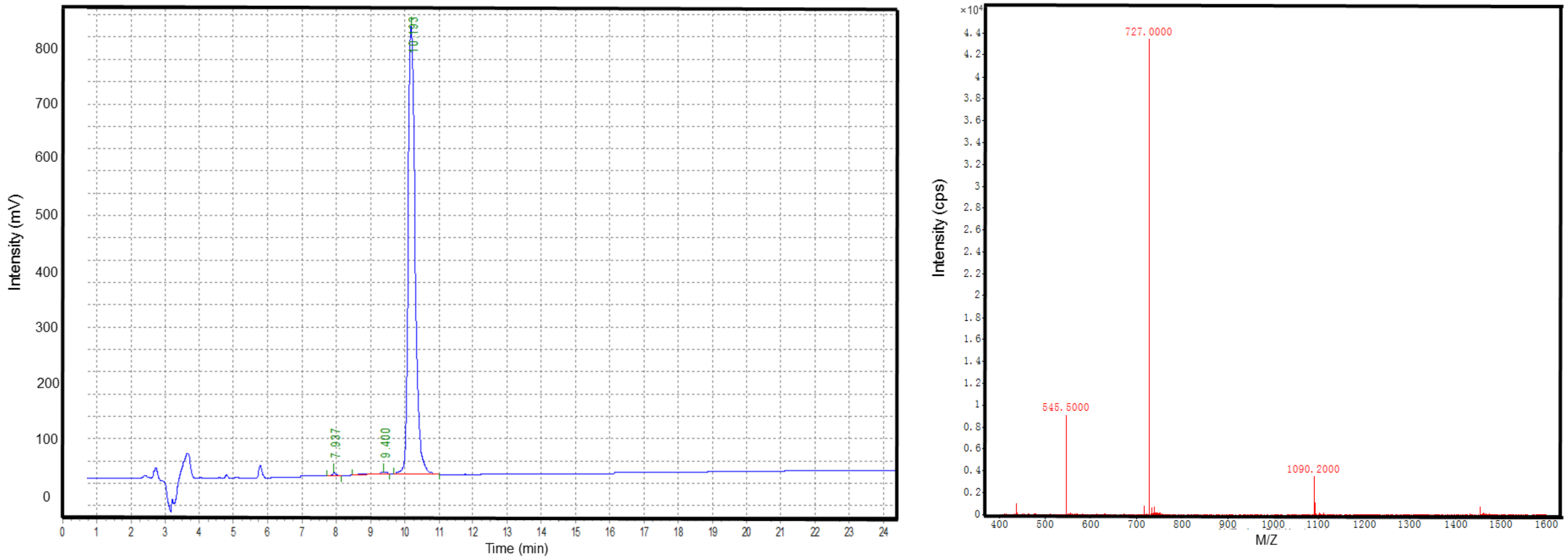

Supplementary Fig. 7. Representative analytical HPLC chromatogram (left) and mass spectra (right) of 5-FAM-labelled scrambled peptide 1 (FAM-SP1). Experimental details were the same as in Supplementary Fig. 1. Panel (a) shows a representative analytical HPLC chromatogram of 5-FAM-labelled scrambled peptide 1. Panel (b) is a typical representative mass spectrum of scrambled peptide 1. The peaks in the mass spectrum represent charged particles with mass-to-charge ratios of $(M+4H)^{4+}$ (545.5000), $(M+3H)^{3+}$ (727.0000) and $(M+2H)^{2+}$ (1090.2000). Repeated twice with different preparations of FAM-SP1 with identical results.

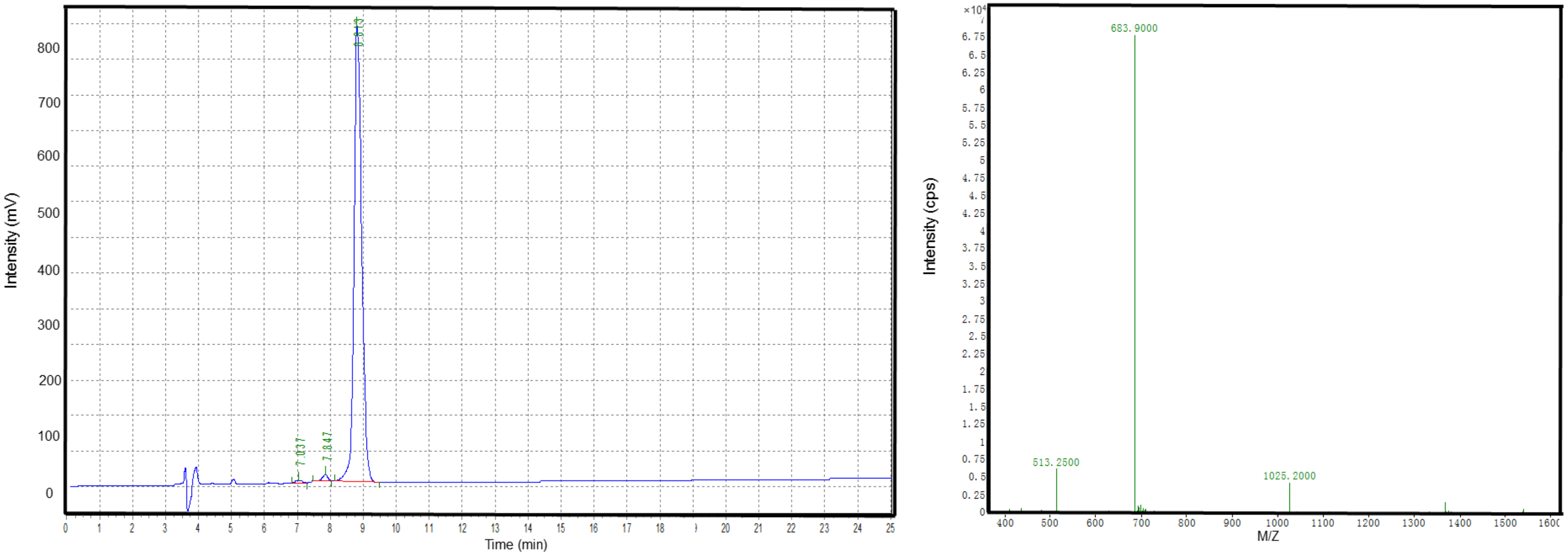

Supplementary Fig. 8. Representative analytical HPLC chromatogram (left) and mass spectra (right) of 5-FAM-labelled scrambled peptide 2 (FAM-SP2). Experimental details were the same as in Supplementary Fig. 1. Panel (a) shows a representative analytical HPLC chromatogram of 5-FAM-labelled scrambled peptide 2. Panel (b) is a typical representative mass spectrum of scrambled peptide 2. The peaks in the mass spectrum represent charged particles with mass-to-charge ratios of $(M+4H)^{4+}$ (513.2500), $(M+3H)^{3+}$ (683.9000) and $(M+2H)^{2+}$ (1025.2000). Repeated twice with different preparations of FAM-SP2 with identical results.

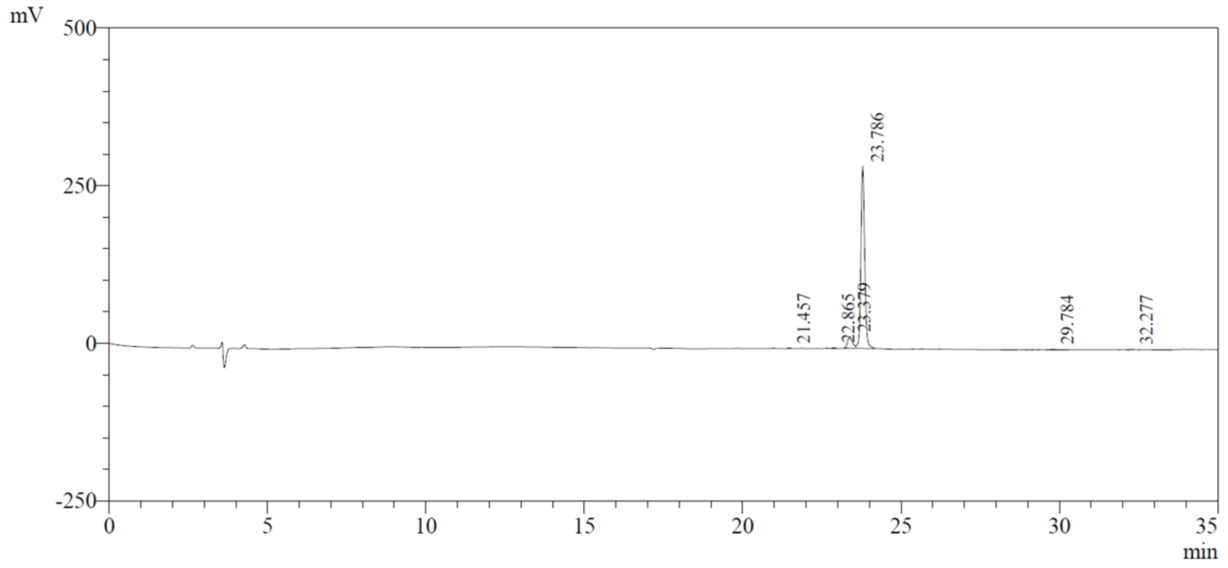

mV
500
250
0
-250
23.786
21.457
22.865
23.379
29.784
32.277
0
5
10
15
20
25
30
35
min

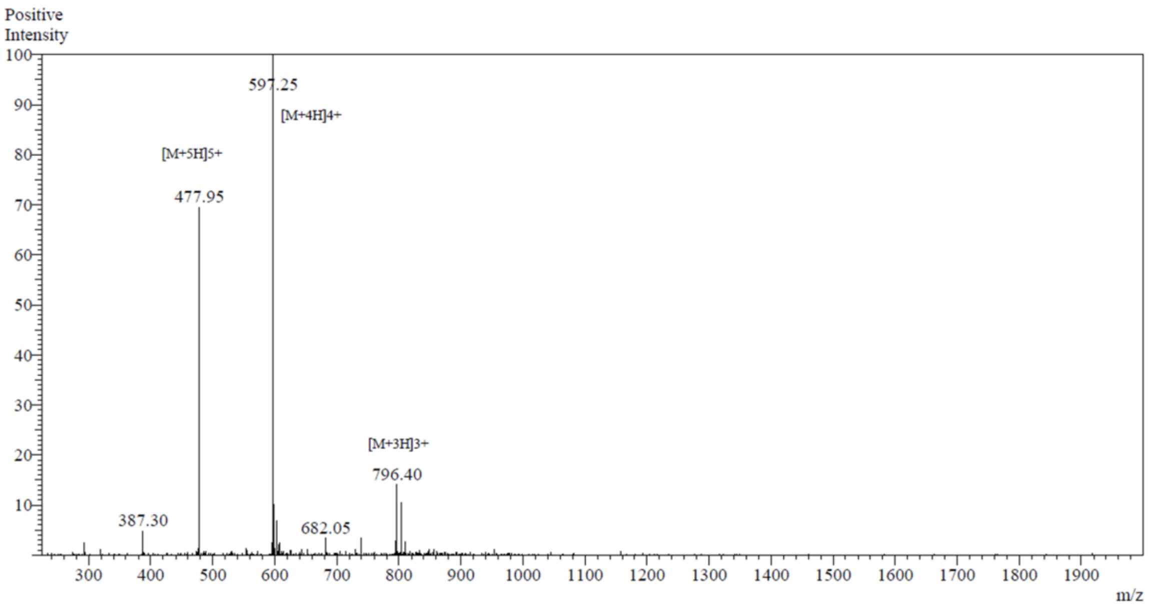

Positive
Intensity
597.25
[M+4H]4+
[M+5H]5+
477.95
[M+3H]3+
796.40
387.30
682.05
m/z

Supplementary Fig. 9. Representative analytical HPLC chromatogram (top) and mass spectra (bottom) of Cy5.5-CGY conjugate. Peptide synthesis is described in Figure S1. For Cy5.5 conjugation, three equivalents of Cy5.5-NHS ester in DMF were coupled with –NH_2 group of peptide by adding ten equivalents of DIEA for 20 min, then 0.1 equivalents of 4-dimethylaminopyridine (DMAP) was added to the reaction vessel and the reaction was allowed for 8 h with light protection. After Cy5.5 conjugation, cleavage of the peptides from the resin was carried out with a mixture of trifluoroacetic acid (TFA, Merck), triisopropylsilane (Tri, Merck), 1,2-ethanedithiol (EDT, Merck) and deionized water in a volume ratio of 95:1:2:2 for 2 h. The solution was concentrated by rotary evaporation, followed by precipitation in cold diethyl ether (Sigma-Aldrich). The crude peptide was collected by filtration, dried under vacuum and further purified using preparative high-performance liquid chromatography (HPLC) (Shimadzu LC-8A) equipped with a C18 column (Grace™ Vydac™, 10×250 mm). The mobile phase was composed of HPLC grade water containing 0.1% (v/v) TFA and acetonitrile containing 0.1% (v/v) TFA and the volume percentage of acetonitrile was gradually increased from 5 to 95% at 45 min, at a flow rate of 10 $mLmin^{-1}$. Top: Shows a representative analytical HPLC chromatogram of Cy5.5-CGY. Bottom: (b) Is a typical representative mass spectrum of FAM-CGY. The peaks in the mass spectrum represent charged particles with mass-to-charge ratios of $(M+5H)^{5+}$ (477.95), $(M+4H)^{4+}$ (597.25) and $(M+3H)^{3+}$ (796.40). The peptide was dissolved in water/acetonitrile (1:1) and injected at 0.2 $mLmin^{-1}$ into the mass spectrometer. Repeated three times with different preparations of Cy5.5-CGY and with identical results.

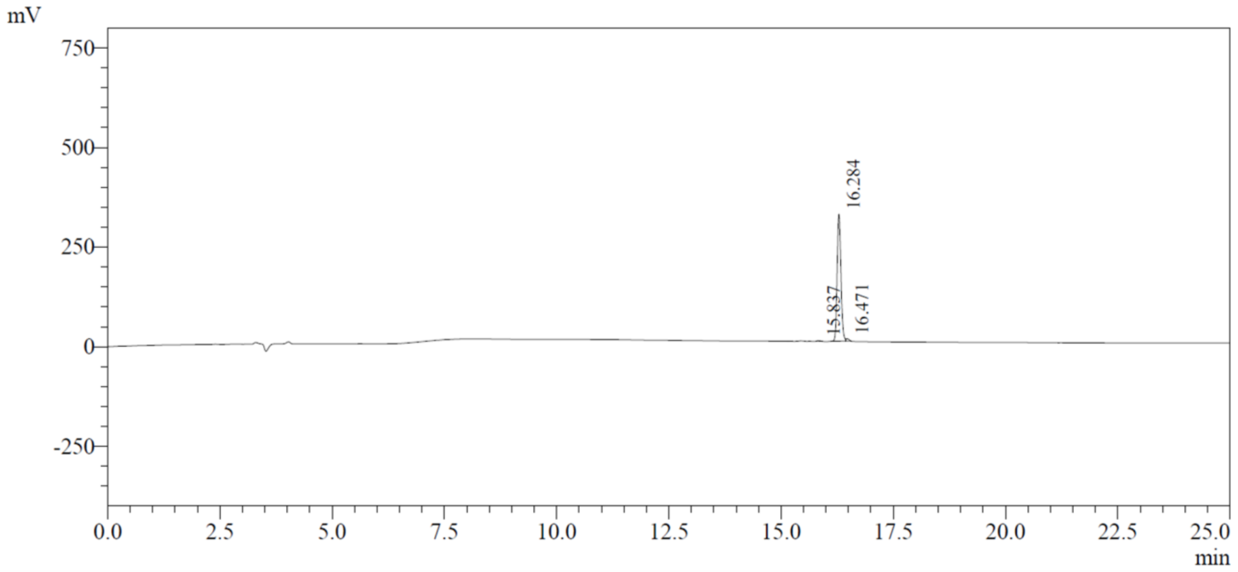
mV
750
500
250
0
-250
16.284
15.837
16.471
0.0
2.5
5.0
7.5
10.0
12.5
15.0
17.5
20.0
22.5
25.0
min

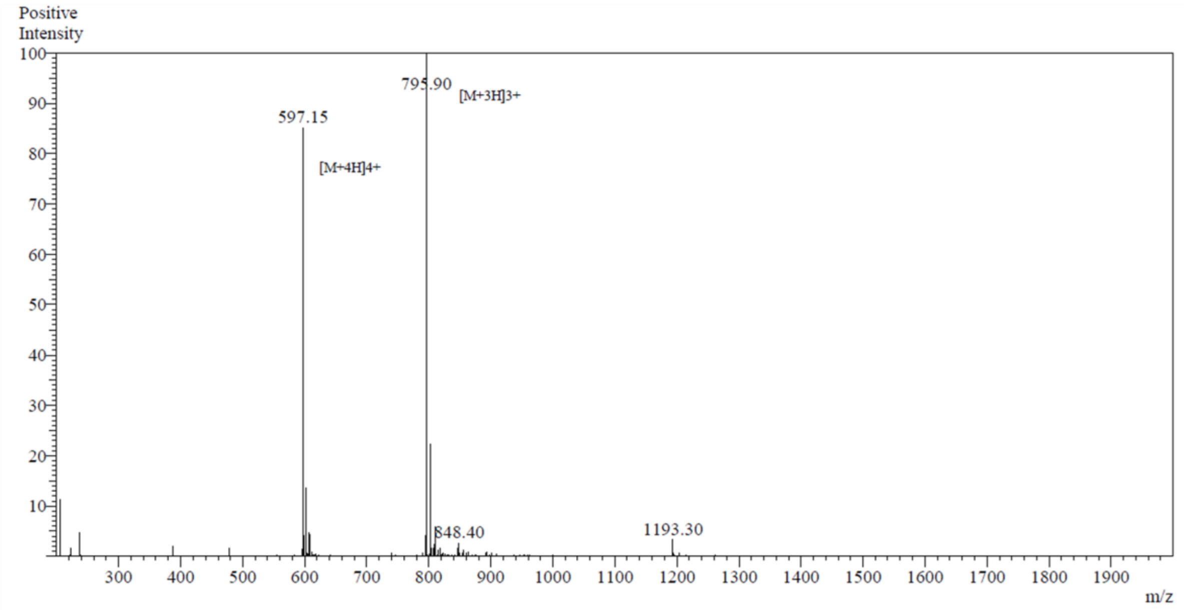
Positive Intensity
795.90
[M+3H]3+
597.15
[M+4H]4+
848.40
1193.30
m/z

Supplementary Fig. 10. Representative analytical HPLC chromatogram (top) and mass spectra (bottom) of Cy5.5-labelled scrambled peptide. The peptide sequence is stated in Table 1. The procedures for peptide synthesis, labelling and purifications are described in Supplementary Fig. 1 & 8. Top: Shows a representative analytical HPLC chromatogram of Cy5.5-scrambled peptide. Bottom: (b) Is a typical representative mass spectrum of Cy5.5-scrambled peptide. The peaks in the mass spectrum represent charged particles with mass-to-charge ratios of $(M+4H)^{4+}$ (597.15), $(M+3H)^{3+}$ (795.90) and $(M+2H)^{2+}$ (1193.30). The peptide was dissolved in water/acetonitrile (1:1) and injected at 0.2 $mLmin^{-1}$ into the mass spectrometer. The experiments were repeated three times with different preparations of Cy5.5-scrambled peptide and with identical results.

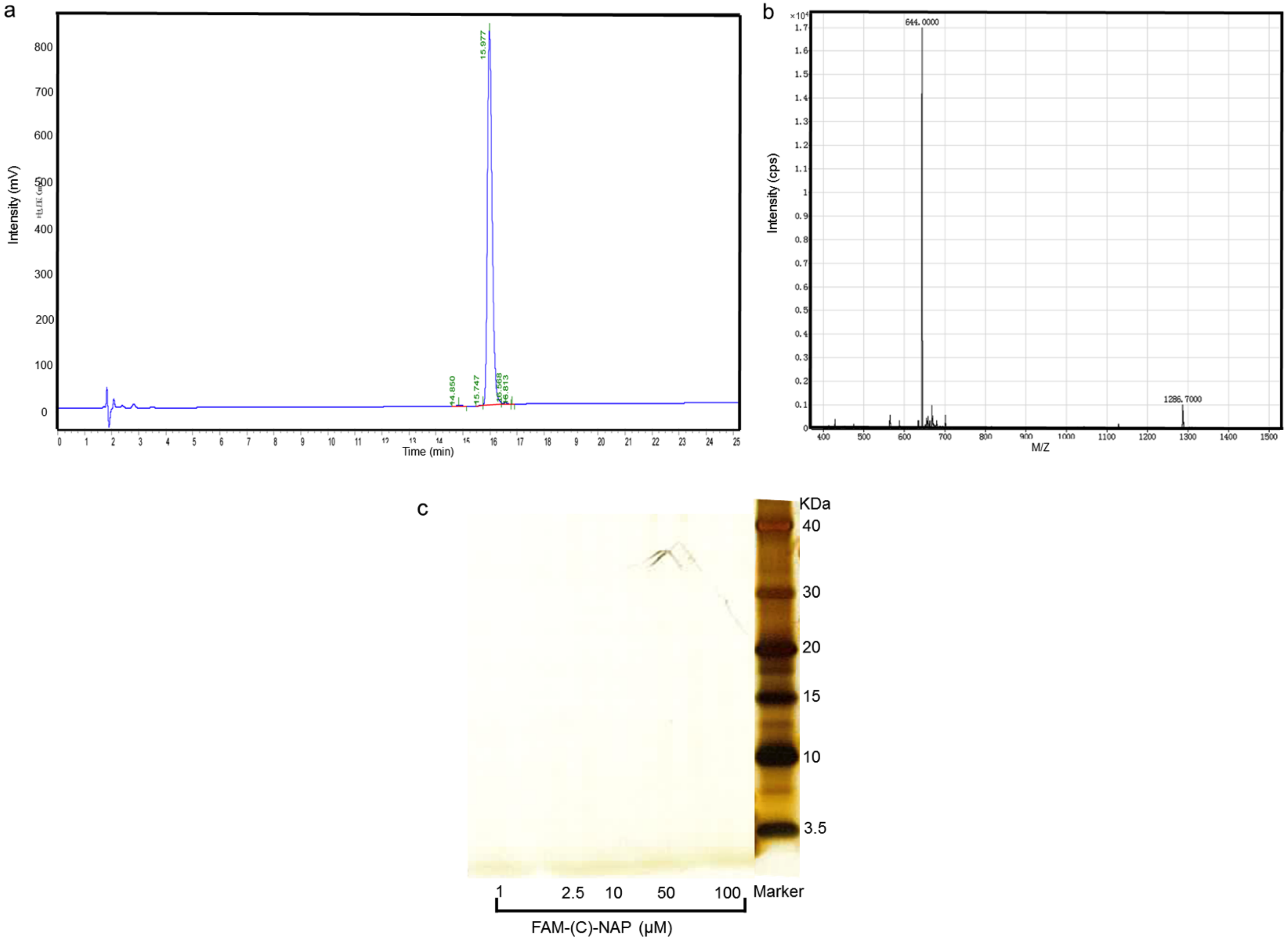

Supplementary Fig. 11. Representative analytical HPLC chromatogram (a), mass spectra (b) and non-reducing SDS PAGE profile (c) of FAM-(C)-NAP peptide. The sequence of the FAM-(C)-NAP peptide is FAM-Cys-Asn-Ala-Pro-Val-Ser-Ile-Pro-Gln and the conjugate has

a molecular mass of 1286.38 $gmol^{-1}$. Details for HPLC procedures were the same as in Supplementary Fig. 1. (a) Shows a representative analytical HPLC chromatogram of FAM-(C)-NAP. (b) Is a typical representative mass spectrum of FAM(C)-NAP. The peaks in the mass spectrum represent charged particles with mass-to-charge ratios of $(M+2H)^{2+}$ (644.9000) and $(M+H)^{+}$ (1286.7000). (c) Non-reducing SDS-PAGE of FAM-(C)-NAP at different concentrations showing no oligomer formation. The experiments were repeated twice with the same peptide preparation.

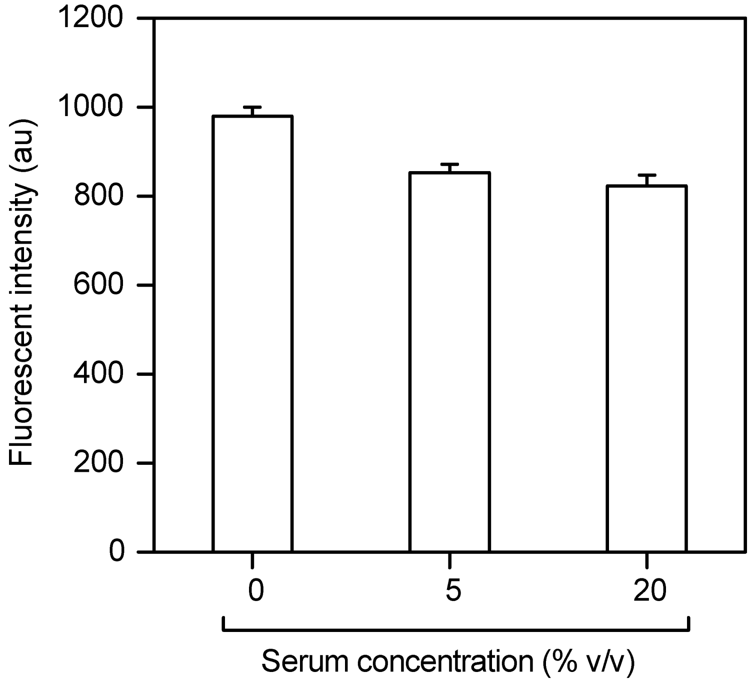

Supplementary Fig. 12. The effect of serum concentration on FAM-CGY assembly uptake by hCMEC/D3 cells. Cells (in triplicate incubations) were exposed to 5 µM FAM-CGY NLCs in the presence of increasing concentration of foetal bovine serum for 16 h at 37ºC. The median cell fluorescence intensity was obtained by flow cytometry of 10,000 analysed cells in a typical experiment. The result represents mean values of the three runs ± s.d. There were no statistical differences in incubations with serum compared with the control incubation (0% v/v serum).

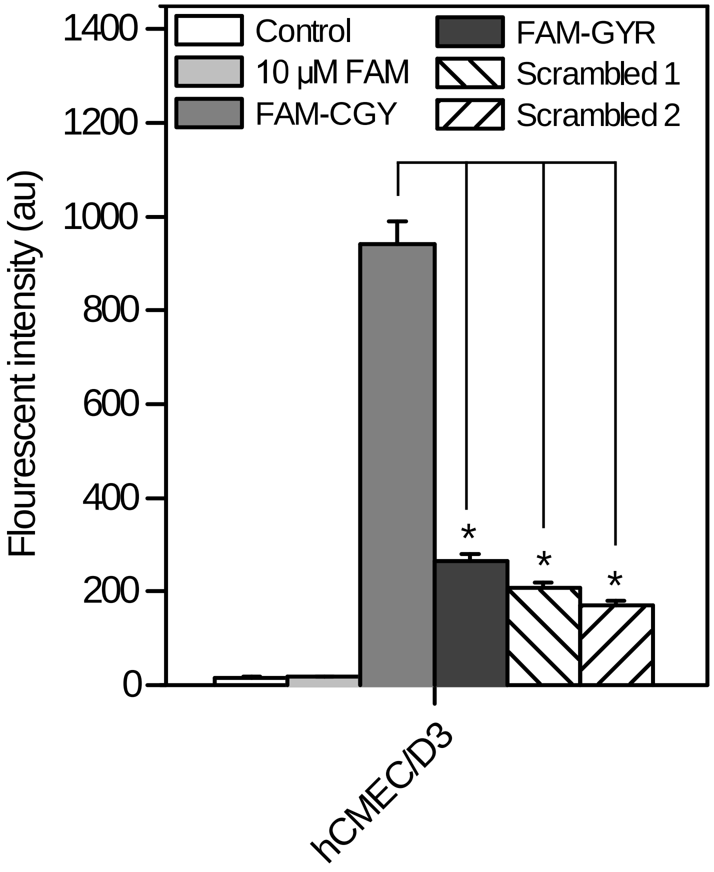

Supplementary Fig. 13. Comparison of FAM-CGY NLC uptake and FAM-CGY analogues by hCMEC/D3 cells. Sequences of all analogues are stated in Table 1. The final concentration of each peptide was 5 µM. All preparations were incubated with cells for 24 h at 37ºC and median cell fluorescence intensity in each typical experiment was obtained by flow cytometry of 10,000 analysed cells. Cell incubations were done in triplicate and each experiment was repeated three times. Results represent mean values of the three experiments ± s.d. *p<0.05, non-paired two-sided student t-test compared with FAM-CGY incubation.

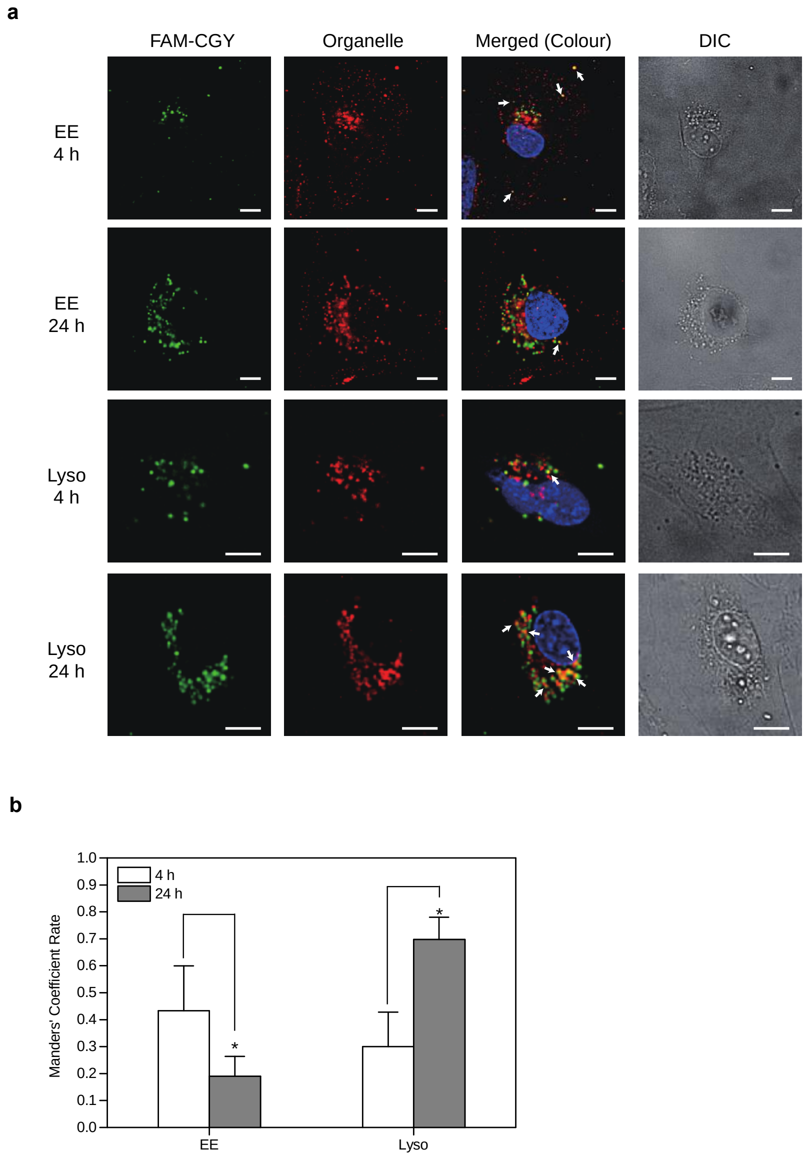

Supplementary Fig. 14. Intracellular co-localization of FAM-CGY NLCs with early endosomes (EE) and lysosomes (Lyso). (a) Live intracellular trafficking of FAM-CGY NLCs (5 µM) in EE and Lyso at 4 and 24 h in hCMEC/D3 cells. Blue, Hoechest 33342-stained nuclei. Images show FAM-CGY NLCs (green) co-localize with EE and Lyso (organelles are in red). Both single and overlap fluorescence channels are shown. DIC images were taken simultaneously to show morphological changes and fluorescence positioning. Insert bar = 20 µm. Images are from a typical experiment (five separate experiments were performed). (b) Mander's overlap coefficient after image analysis showing the extent of FAM-CGY NLC-derived fluorescence overlap in EE and Lyso compartments

in hCMEC/D3 cells. Minimum of 20 random cells representing each time-point for both organelles were analysed. *$p<0.05$, non-paired two-sided student t-test.

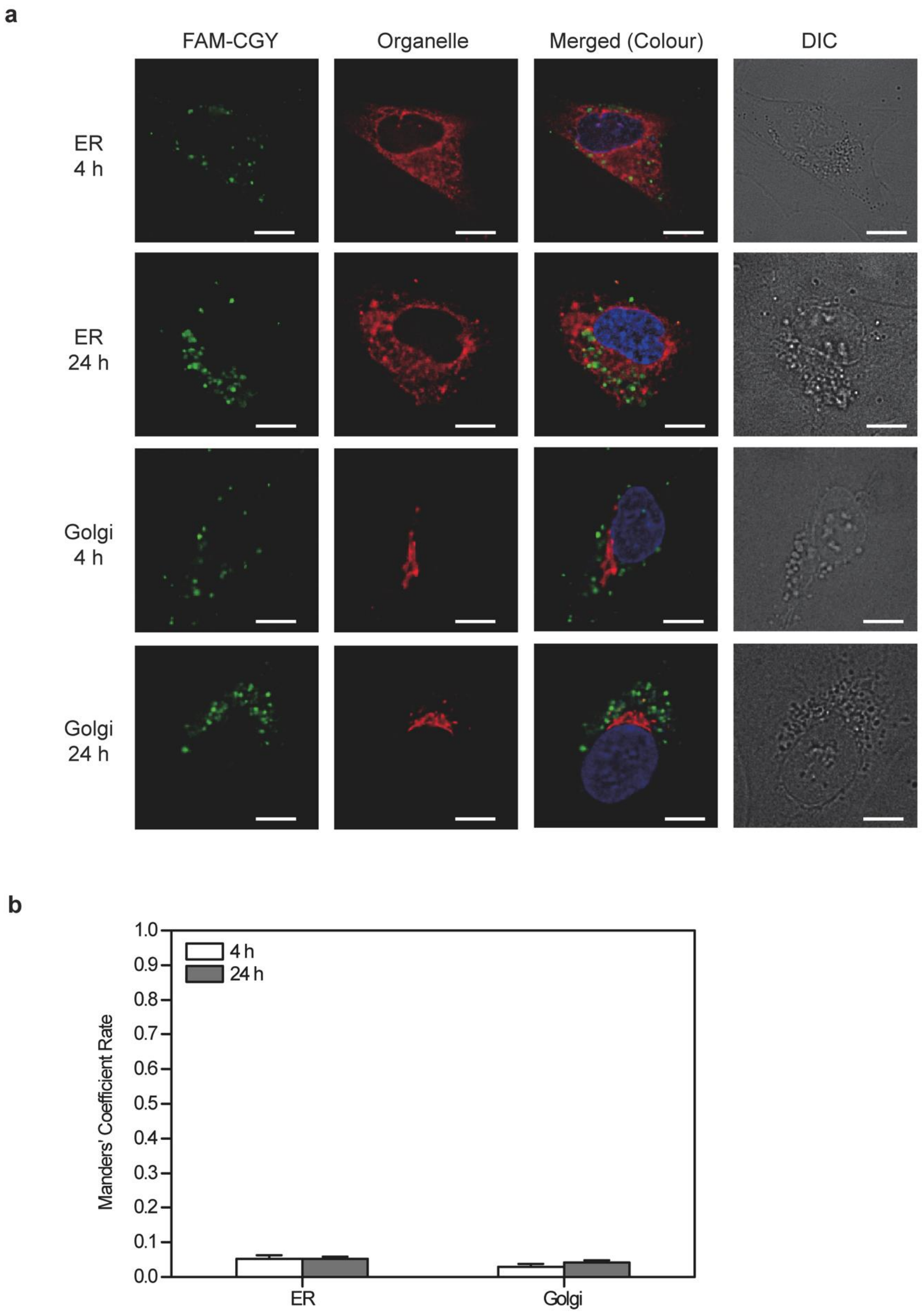

Supplementary Fig. 15. Intracellular co-localization of FAM-CGY NLCs with endoplasmic reticulum (ER) and the Golgi apparatus. (a) Live intracellular trafficking of FAM-CGY NLCs in ER and the Golgi apparatus at 4 and 24 h post FAM-CGY NLC (5 µM) addition to hCMEC/D3 cells. FAM-CGY NLC is green and the organelles (ER and Golgi) are red. Blue, Hoechest 33342-stained nuclei. Both single and overlap fluorescence channels are shown. DIC images were taken

simultaneously to show morphological changes and fluorescence positioning. Insert bar = 20 µm. Images are from a typical experiment (five separate experiments were performed). (b) Mander's overlap coefficient after image analysis showing the extent of FAM-CGY NLC-derived fluorescence overlap in ER and the Golgi apparatus in hCMEC/D3 cells. Minimum of 20 random cells representing each time-point for both organelles were analysed. $p>0.05$, non-paired two-sided student t-test comparing 4 h versus 24 h points in each organelle.

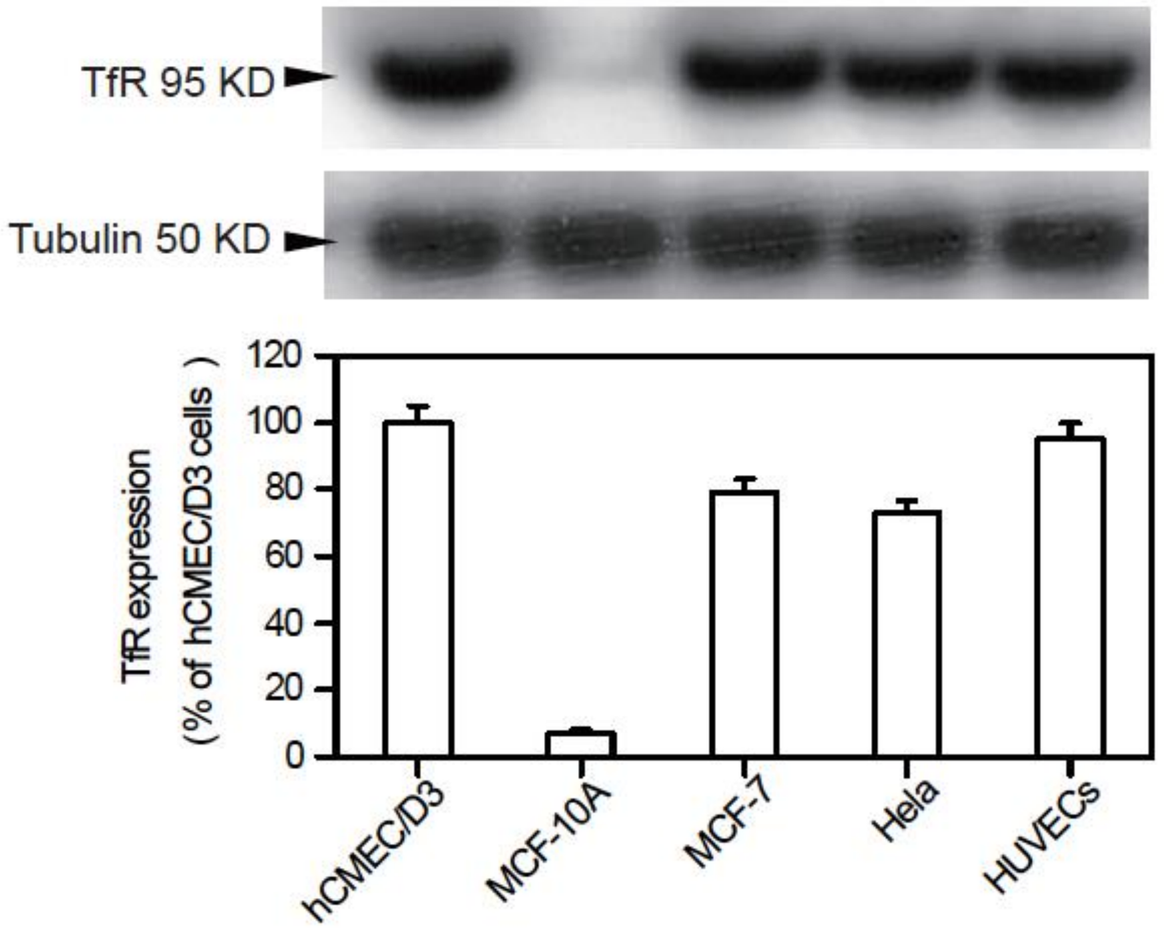

Supplementary Fig. 16. Transferrin receptor (TfR) expression level in different human cells. TfR expression was determined by Western blotting with tubulin serving as internal control reference. The level of TfR expression in hCMEC/D3 cells was taken as 100%. The TfR expression level in other cells is compared relative to hCMEC/D3 cells. MCF-7, Hela cells and human umbilical vein endothelial cells (HUVECs) (as positive control) express TfR in a comparable level to hCMC/D3 cells. MCF-10A shows poor expression of TfR compared to all other cells (p<0.01; non-paired two-sided student t-test). The results are expressed as mean value of three separate experiments ± s.d.

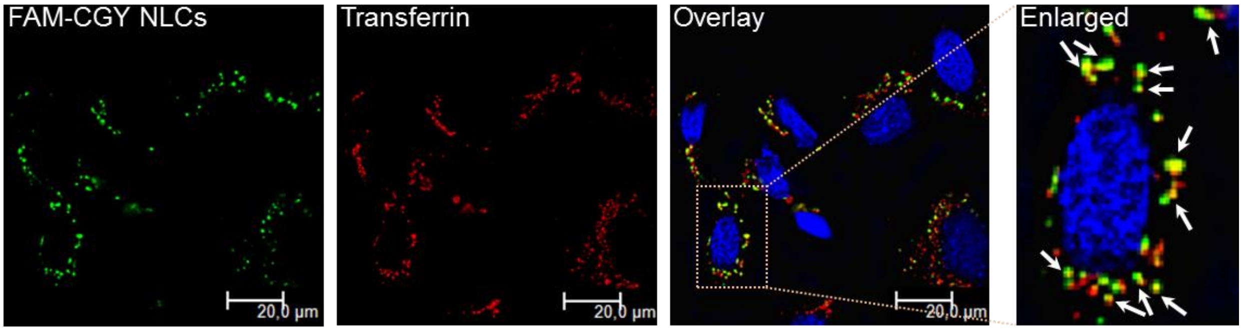

Supplementary Fig. 17. Intracellular co-localization of FAM-CGY NLCs with transferrin. hCMEC/D3 cells were challenged with 5 µM FAM-CGY (green) and 5 µg Texas Red®-Transferrin (red) for 24 h. The cell nucleus was labelled with Hoechest 33342 (blue). bar = 20 µm. Arrows indicated typical fluorescent overlap between FAM-CGY NLCs with transferrin. The experiment was repeated 3 times.

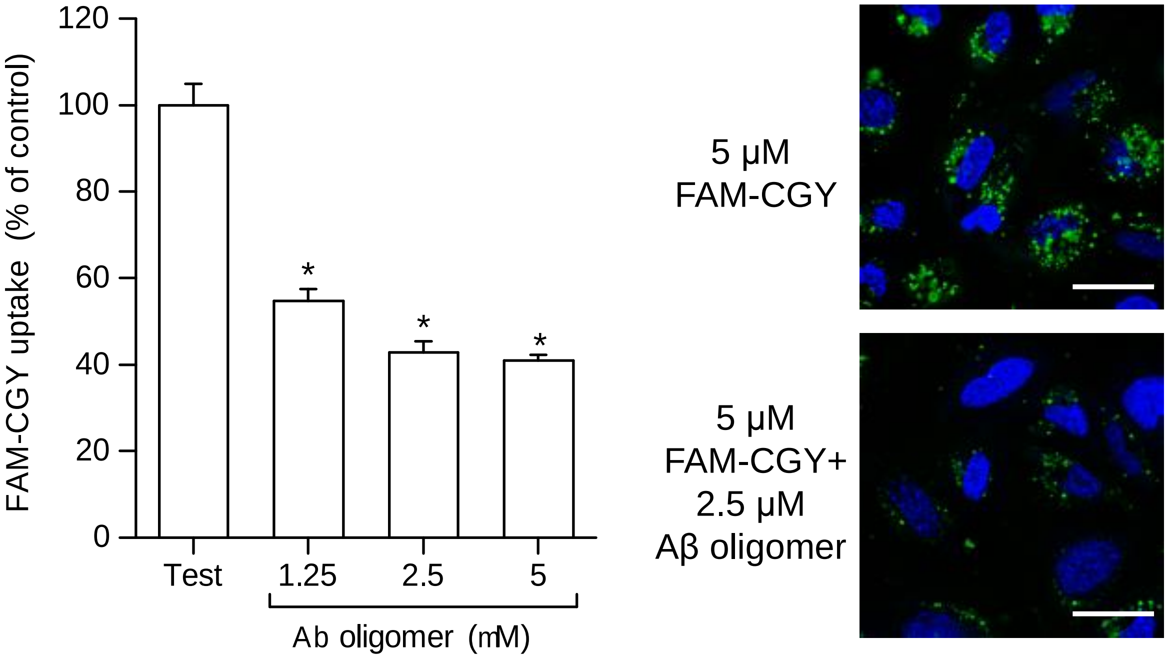

Supplementary Fig. 18. Competition between FAM-CGY NLC and Aβ oligomer uptake by hCMEC/D3 cells. The results show Aβ oligomers compete with the uptake of FAM-CGY NLCs (left panel). A representative live-cell fluorescence microscopy is shown in the right panel. Insert bar = 20 μm. The median cell fluorescence intensity in each typical experiment (n = 3) was obtained by flow cytometry of 10,000 analysed cells. Cell incubations were done in triplicate and each experiment was repeated three times. Results represent mean values of the three experiments ± s.d. *p<0.05, non-paired two-sided student t-test compared with Test incubation (no Aβ oligomers).

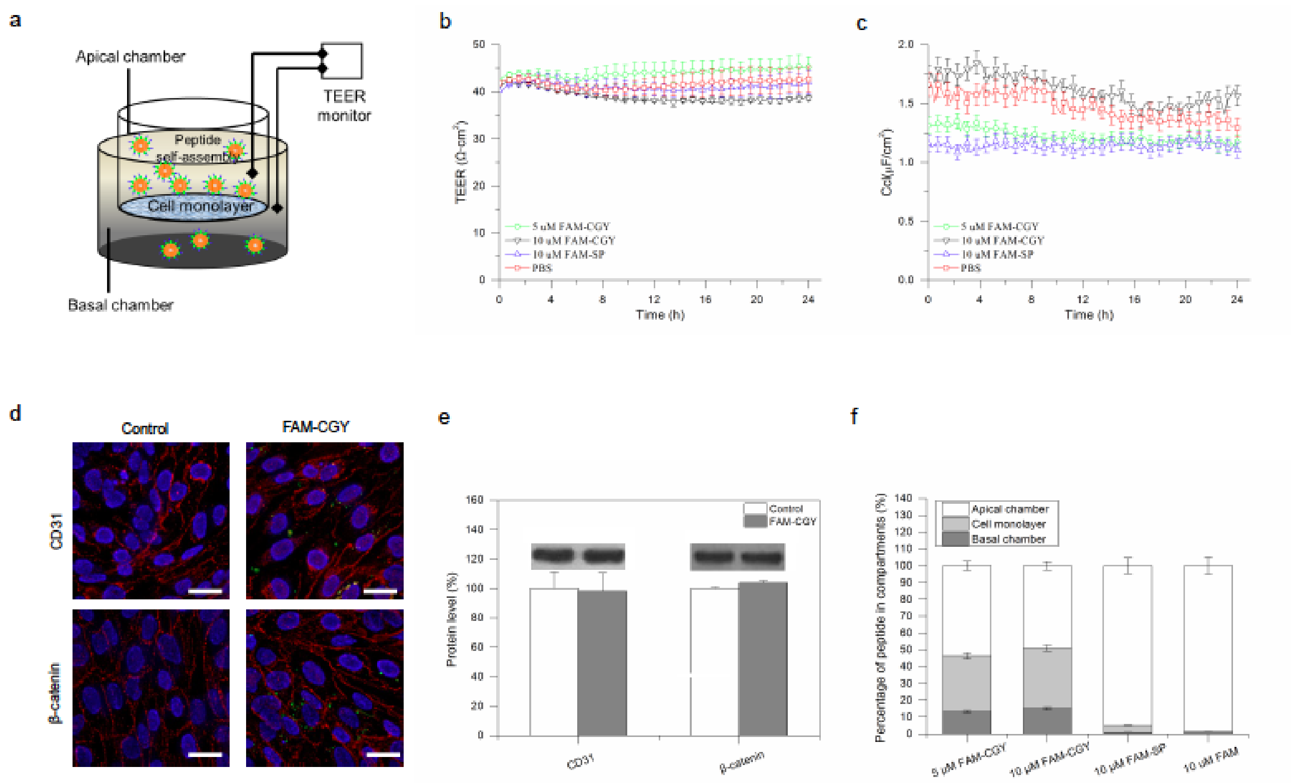

Supplementary Fig. 19. Charcaterisation of *in vitro* model of the blood-brain-barrier, NLC uptake and translocation. (a) Schematic illustration of the transwell model of the human BBB model. (b) Trans-endothelial electrical resistance (TEER) and cell layer capacitance (C) (which reflects the membrane surface area) measurements with a CellZscope. TEER values were peaked on day 7 (~ 40Ωcm^{-2}) in transwell inserts and longer culture of cells (up to 10 days) did not improve TEER and formation of multiple cell-layers was sometime observed from day 7 onward. Again, at day 7 the cell layer capacitance was below 2 μFcm^{-2} (compared with ~ 10 μFcm^{-2} in empty inserts). TEER values and its corresponding capacitance were recorded after the introduction of peptide for 24 h. (d) & (e) Staining and quantification of endothelial cells (CD31) and adheren β-catenin markers confirming the presence of a functional tight junction before and after NLC (5 μM) challenge. Green: FAM-CGY, Red: CD31 or β-catenin, Blue: Nucleus. Bar = 20 μm. (f) Transport efficacy of peptide in in vitro BBB monolayer. The fluorophor FAM alone was used as reference. In (b), (c), (e) and (f) the results represent mean values of 3 separate experiments ± s.d.

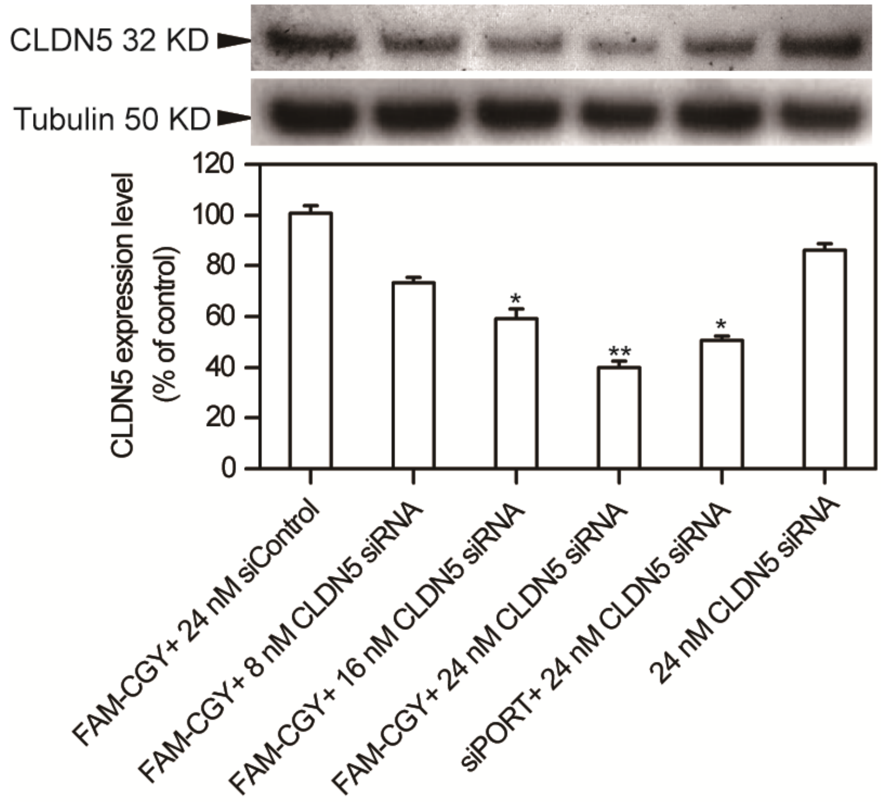

Supplementary Fig. 20. Down regulation of Claudin-5 (CLDN5) expression by different FAM-CGY/CLDN5 siRNA complexes. FAM-CGY (5 µM final concentration)/CLDN5-specific siRNA assemblies effectively down regulates CLDN5 in hCMEC/D3 cells as determined by Western blotting. Down regulation is also compared with CLDN5-specific siRNA alone and when delivered with the commercial transfectant siPORT. CLDN5 expression was measured 72 h after transfection. The results represent mean values of three separate experiments ± s.d. *p<0.05 and **p<0.01, non-paired two-sided student t-test compared with respective siControl (control siRNA).

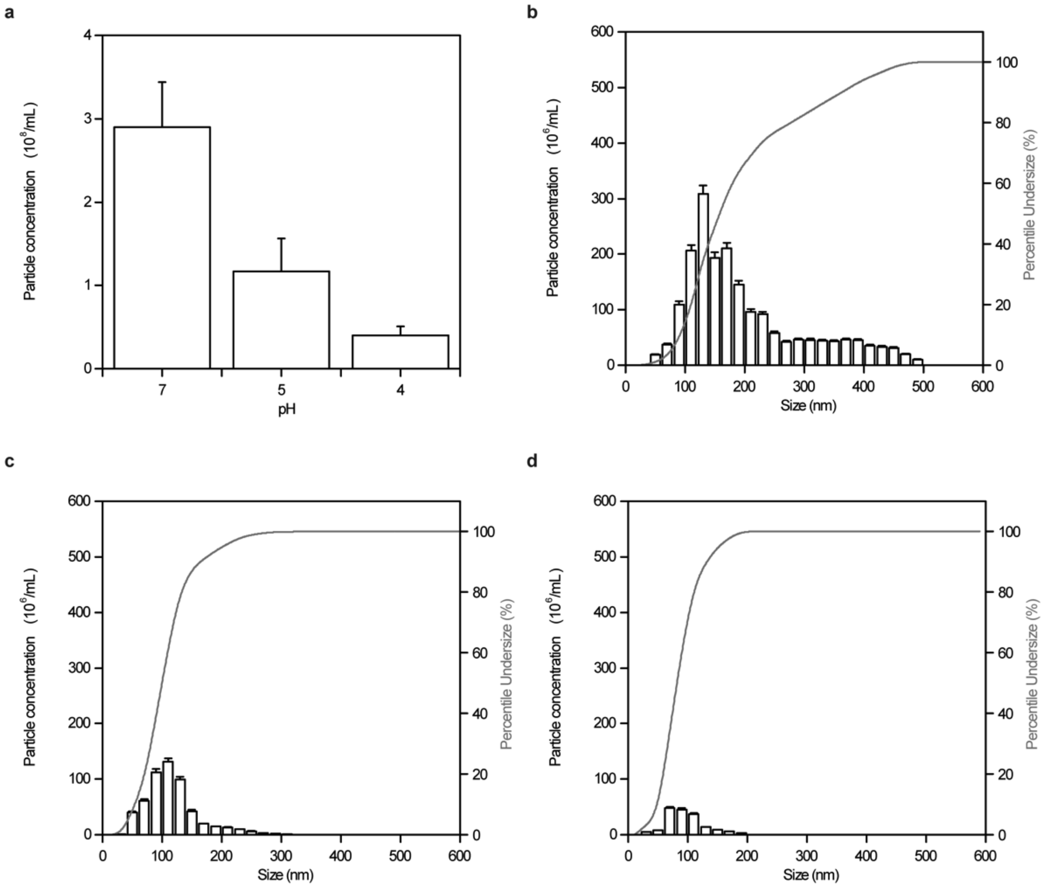

Supplementary Fig. 21. The effect of pH in suspending medium on FAM-CGY NLC concentration and size distribution. Nanoparticle Tracking Analysis (NTA) was used to determine both particle size distribution and concentration. For details see Methodology. (a) The effect of pH on mean FAM-CGY NLC particle concentration (n = 3 determinations at 37ºC). The results are expressed as the mean value ± s.d. (b), (c) and (d) represent FAM-CGY NLC size distribution as a function of particle concentration at pH 7.0, 5.0 and 4.0, respectively. The results in each panel are mean values ± s.d. from three separate determinations. Decreasing pH from 7.0 to 4.0 decreases the overall concentration of FAM-CGY particles (compare panel (a) with panels (b) and (c)). This is due to disassembly of FAM-CGY particles to peptide monomer or oligomers. FAM-CGY working concentration (5 µM) was made in 10 mM HEPES buffer and pH values were adjusted with 1 M HCl or 4 M NaOH.

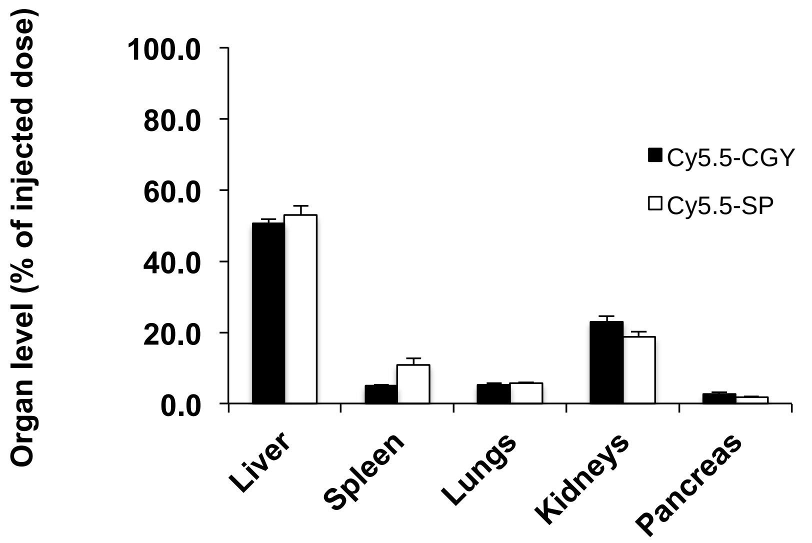

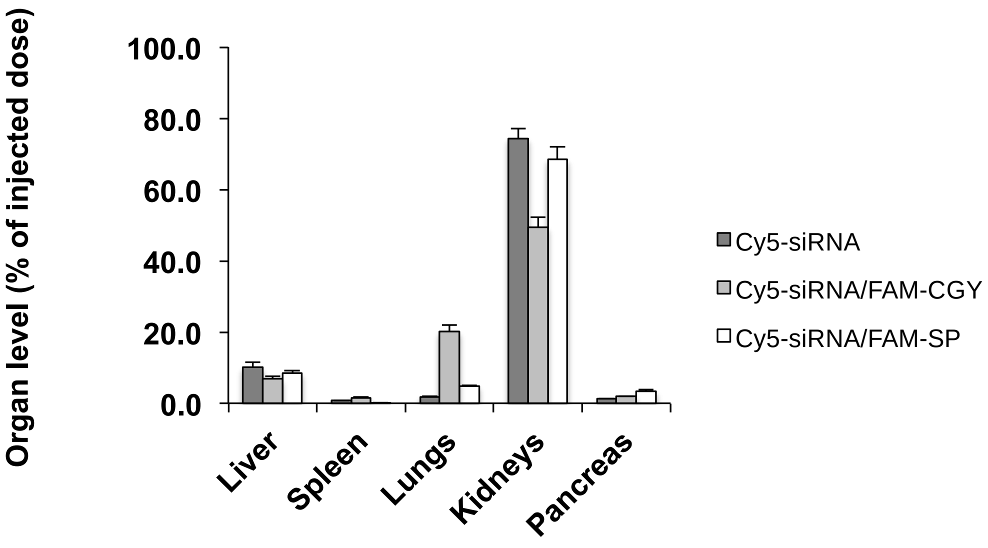

Supplementary Fig. 22. Biodistribution of intravenously injected NLCs and their assemblies with siRNA at 4 h post injection. Groups of male and females (50:50 distribution) ICR mice (6–8 weeks old, body weight 20–26 g) were randomly selected (n = 3 per group) and injected with Cy5.5-CGY

NLCs, Cy5.5-CGY scramble peptide (Cy5.5-SP), naked Cy5-siRNA, Cy5-siRNA/FAM-CGY complexes, and Cy5-siRNA/FAM-CGY scramble peptide complexes via tail vein. The final concentration of the peptide conjugate was 10 μM and that of siRNA was 48 nM based on blood volume calculation equivalent to 6% of the body weight. At 4 h post administration, mice were sacrificed, and selected organs were collected and stored in liquid nitrogen. The organs were homogenized (FastPrep-24™ 5G Instrument, MP Biomedicals, USA) and the levels of peptide and siRNA were measured in a fluorescence spectrometer (Fluotime 300, Picoquant, Berlin, Germany) using a standard curve. The left panel shows organ distribution of Cy5.5-CGY NLCs and Cy5.5-CGY scrambled peptide. The right panel shows organ distribution of naked Cy5-siRNA and siRNA delivered on complexion with FAM-CGY and FAM-CGY scrambled peptide. The results are mean ± s.e.m (n = 3).

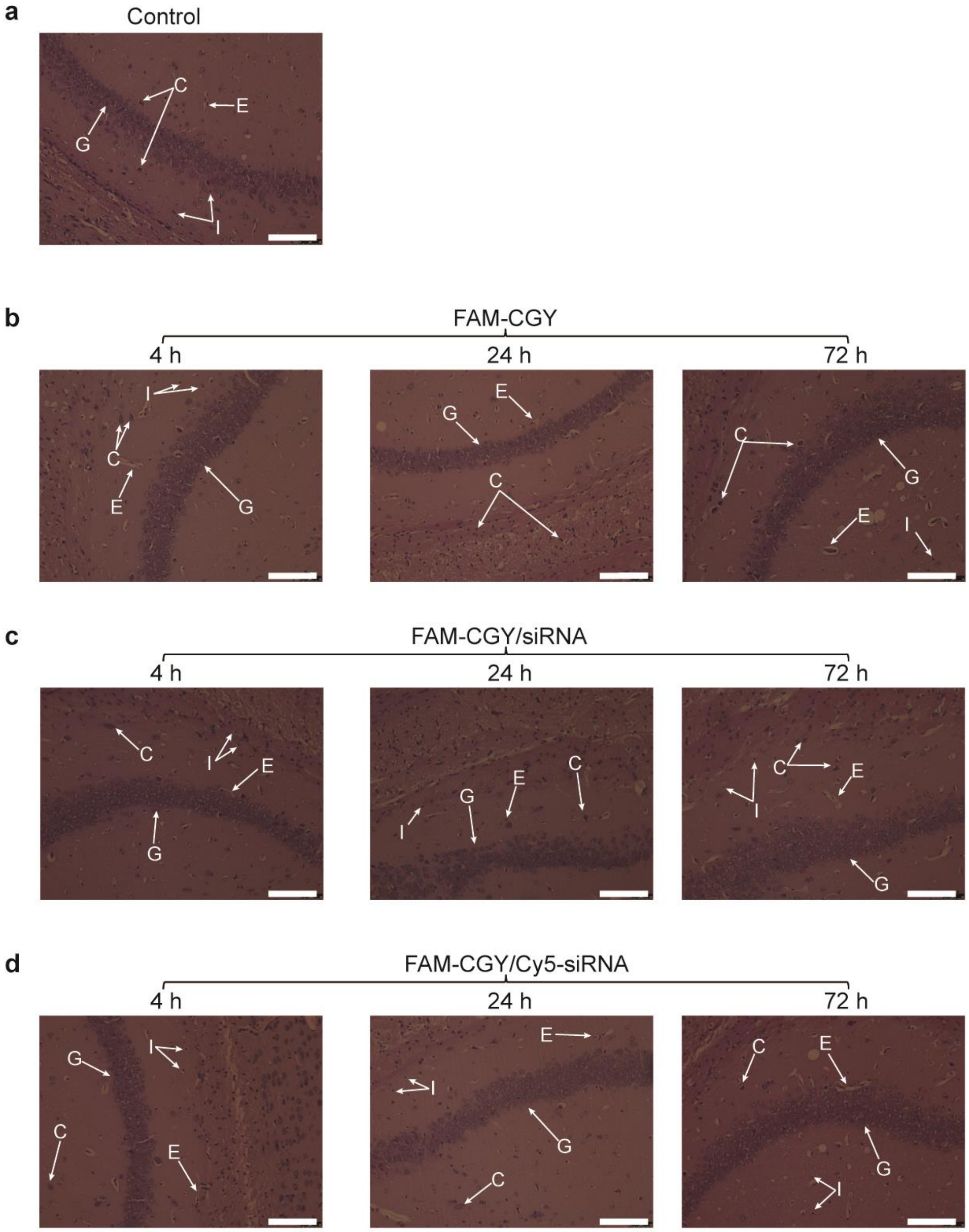

Supplementary Fig. 23. Haematoxylin and eosin-stained sections of hippocampus from mice (n = 3 per group) injected intravenously with saline (control) (a), FAM-CGY (10 μM) (b), FAM-CGY/BACE-1-specific siRNA (10 μM/48 nM) (c) and FAM-CGY/Cy5-siRNA (10 μM/48 nM) (d). Sections were examined at 4, 24 and 72 h post injection. C, G, E and I indicated conical nerve cell, granular cell, vascular endothelial cell and gliacyte, respectively. Scale bar = 100 μm. NLC

treatment does not induce neuronal injury/swelling, cellular liquefaction, necrosis and focal inflammatory cellular infiltration in hippocampus. Experiments were repeated twice with sections from different animals (n = 3).

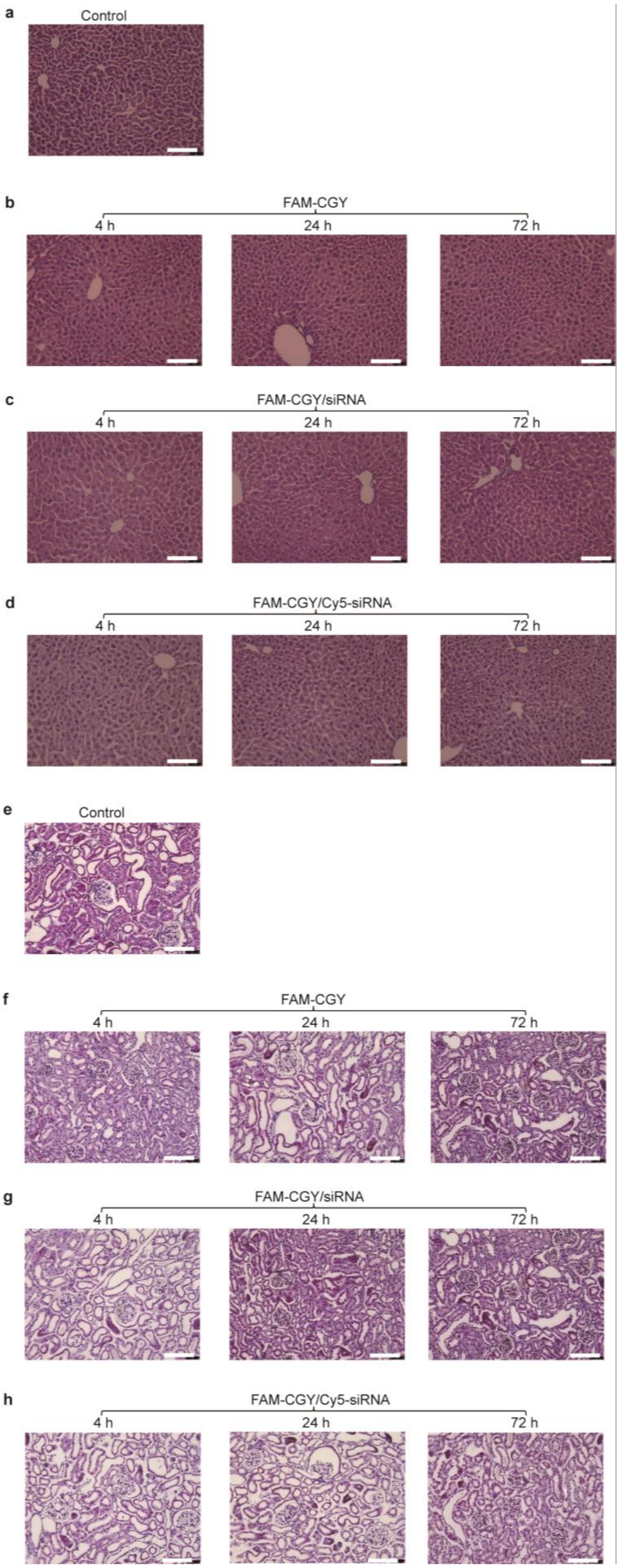
a
Control
b
FAM-CGY
4 h
24 h
72 h
c
FAM-CGY/siRNA
4 h
24 h
72 h
d
FAM-CGY/Cy5-siRNA
4 h
24 h
72 h
e
Control
f
FAM-CGY
4 h
24 h
72 h
g
FAM-CGY/siRNA
4 h
24 h
72 h
h
FAM-CGY/Cy5-siRNA
4 h
24 h
72 h

Supplementary Fig. 24. Haematoxylin and eosin-stained sections of liver (a–d) and kidney (e–h) from mice (n = 3 per group) injected intravenously with saline (control) (a), FAM-CGY (10 μM) (b), FAM-CGY/BACE-1-specific siRNA (10 μM/48 nM) (c) and FAM-CGY/Cy5-siRNA (10 μM/48 nM) (d). Sections were examined at 4, 24 and 72 h post injection. Scale bar = 100 μm. The results show NLC and NLC/siRNA treatment has no detrimental effect on the liver and the kidney and comparable to saline treatment. Experiments were repeated twice with sections from different animals (n = 3).

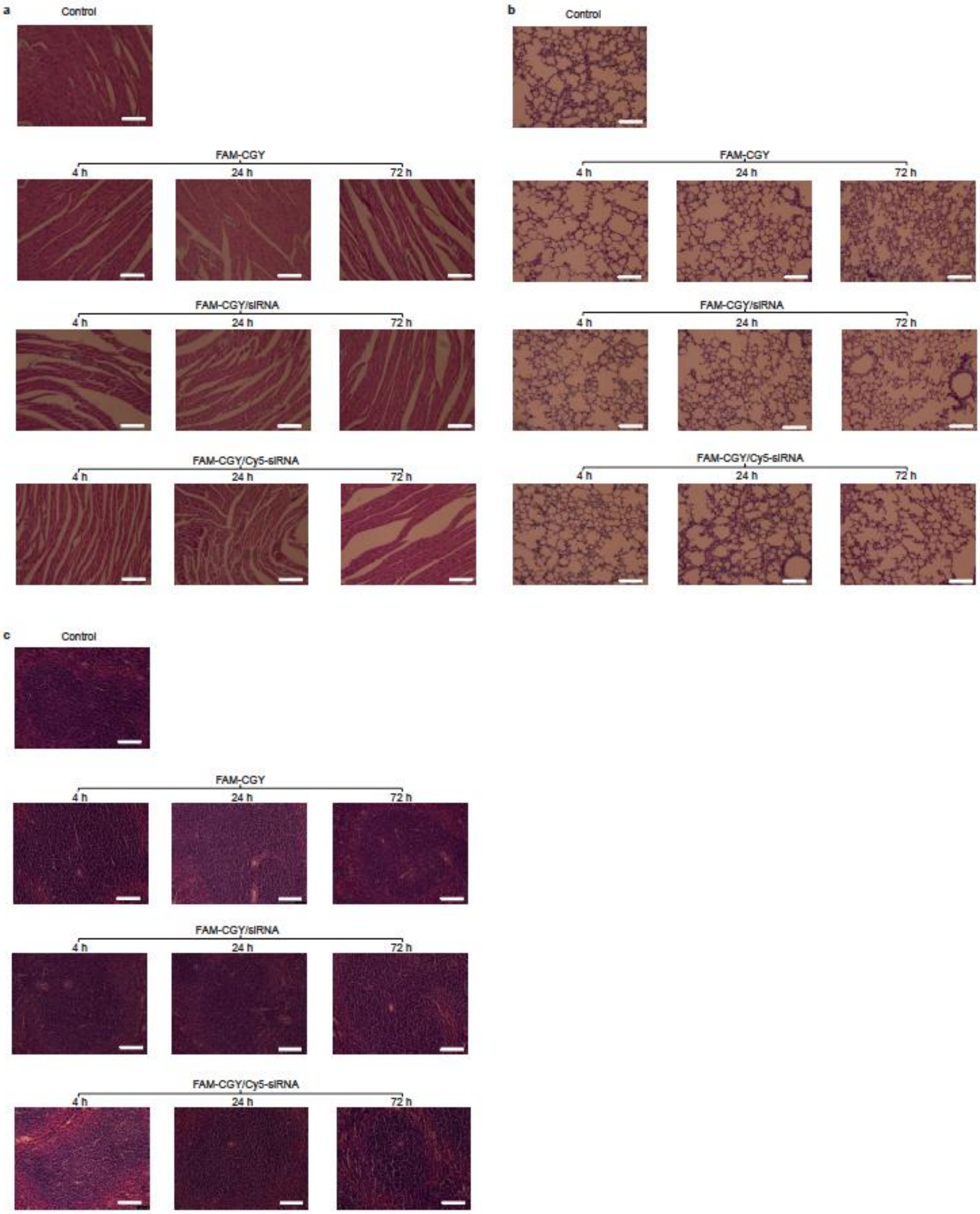

Supplementary Fig. 25. Haematoxylin and eosin-stained sections of heart (a), lungs (b) and spleen (e) from mice (n = 3 per group) injected intravenously with saline (control) (a), FAM-CGY (10 μM)

(b), FAM-CGY/BACE-1-specific siRNA (10 μM/48 nM) (c) and FAM-CGY/Cy5-siRNA (10 μM/48 nM) (d). Sections were examined at 4, 24 and 72 h post injection. Scale bar = 100 μm. The results show NLC and NLC/siRNA treatment has no detrimental effects on the organs and comparable to saline treatment. Experiments were repeated twice with sections from different animals (n = 3).

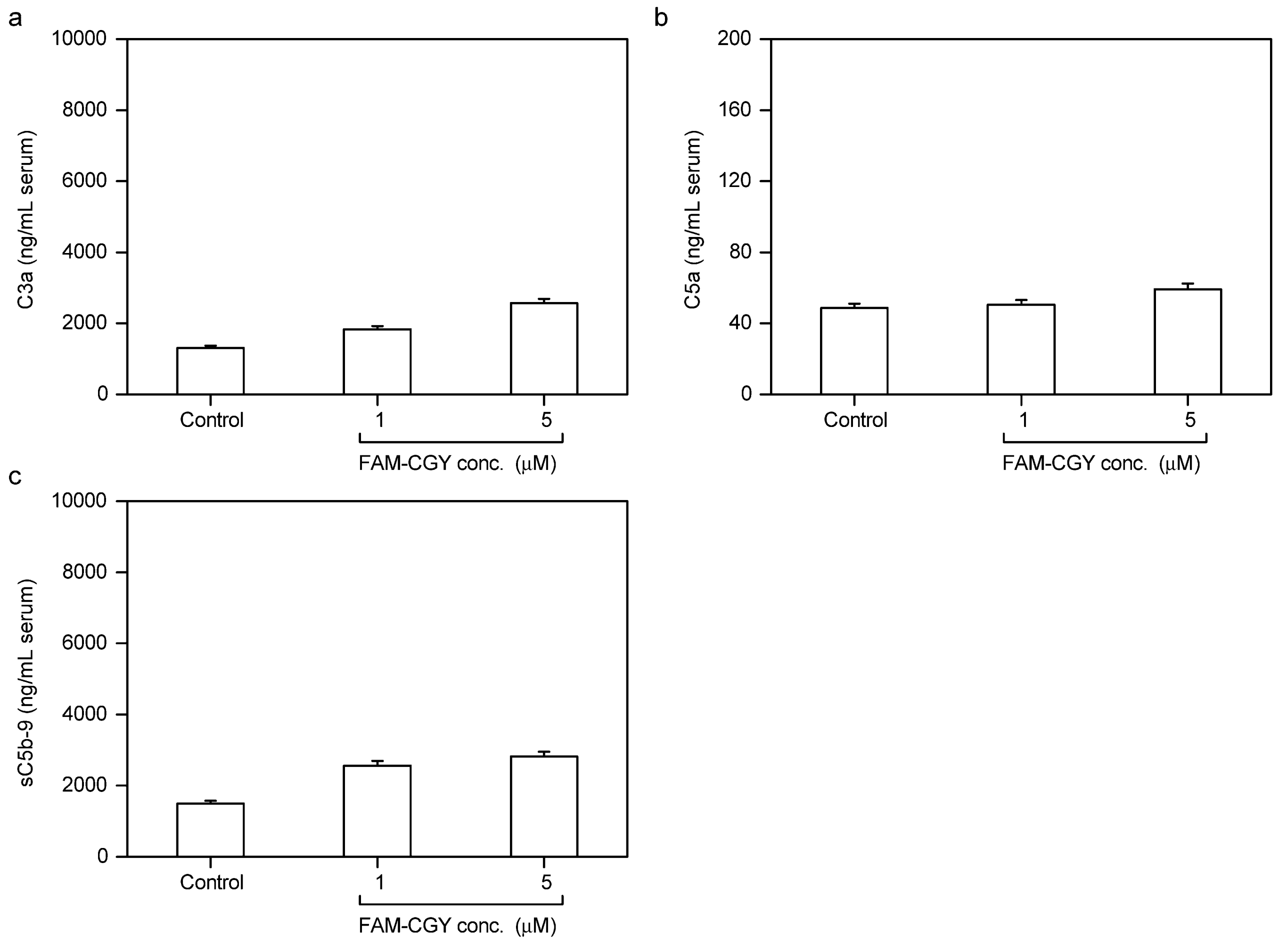

Supplementary Fig. 26. The effect of FAM-CGY on complement activation in human serum. Complement activation was measured by monitoring generation of complement activation products C3a, C5a and sC5b-9. Complement activation was monitored at below and above FAM-CGY critical aggregation concentration. (a) The effect of FAM-CGY on C3a release. (b) The effect of FAM-CGY on C5a release. (c) The effect of FAM-CGY on sC5b-9 generation. FAM-CGY slightly increased levels of C3a and sC5b-9 in serum compared to control background incubation, but these differences were not statistically significant (p>0.05). Zymosan was used as positive control for monitoring complement activation in serum. Zymosan generated 18,133 ± 907 ngmL^{-1} serum C3a, 1553 ± 31 ngmL^{-1} serum C5a and 14,220 ± 721 ngmL^{-1} serum sC5b-9. Incubations were performed in triplicate. The results are expressed as mean ± s.e.m. The experiment was repeated in 3 different sera and the same patterns of complement activation were observed. In each case complement activation was statistically not significant (compared with control; non-paired two-sided student t-test).

Section for original uncropped gel/blot images

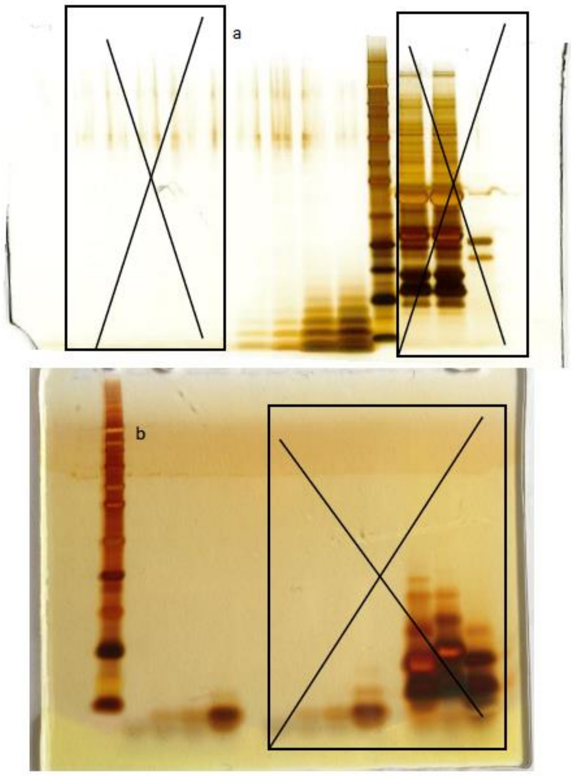

Original uncropped silver-stained SDS-PAGE gel for Fig. 1a and 1b.

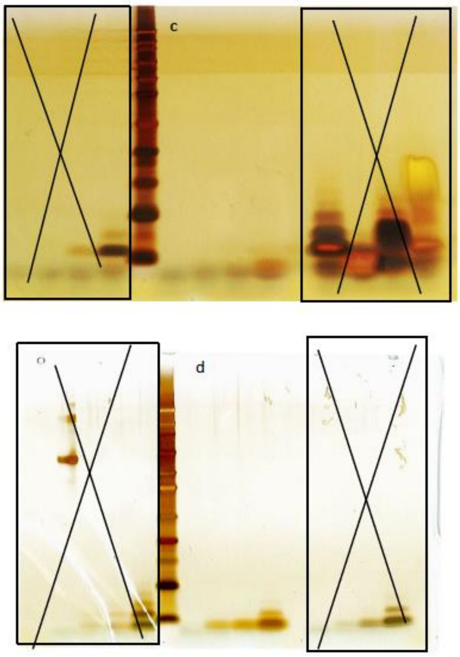

Original uncropped silver-stained SDS-PAGE gel for Fig. 1c and 1d.

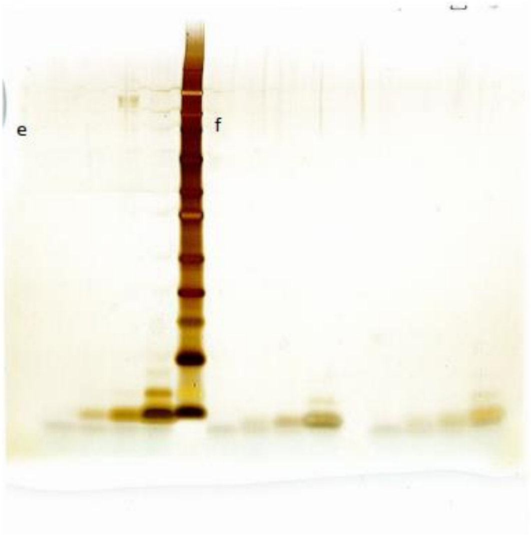

Original uncropped silver-stained SDS-PAGE gel for Fig. 1e and 1f.

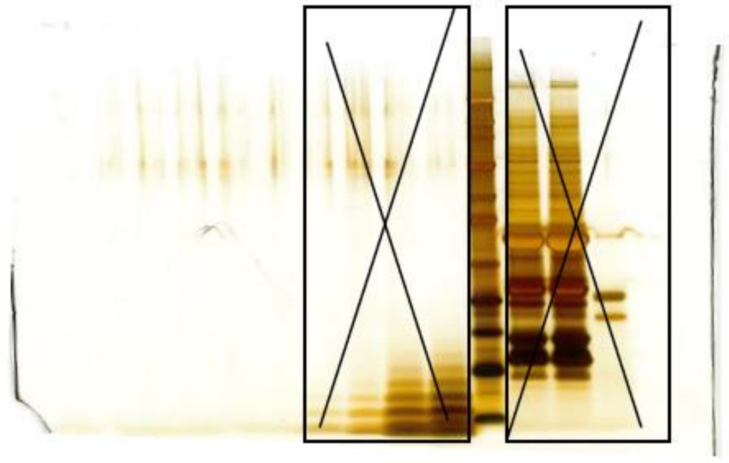

Original uncropped silver-stained SDS-PAGE gel for Supplementary Fig. 11c.

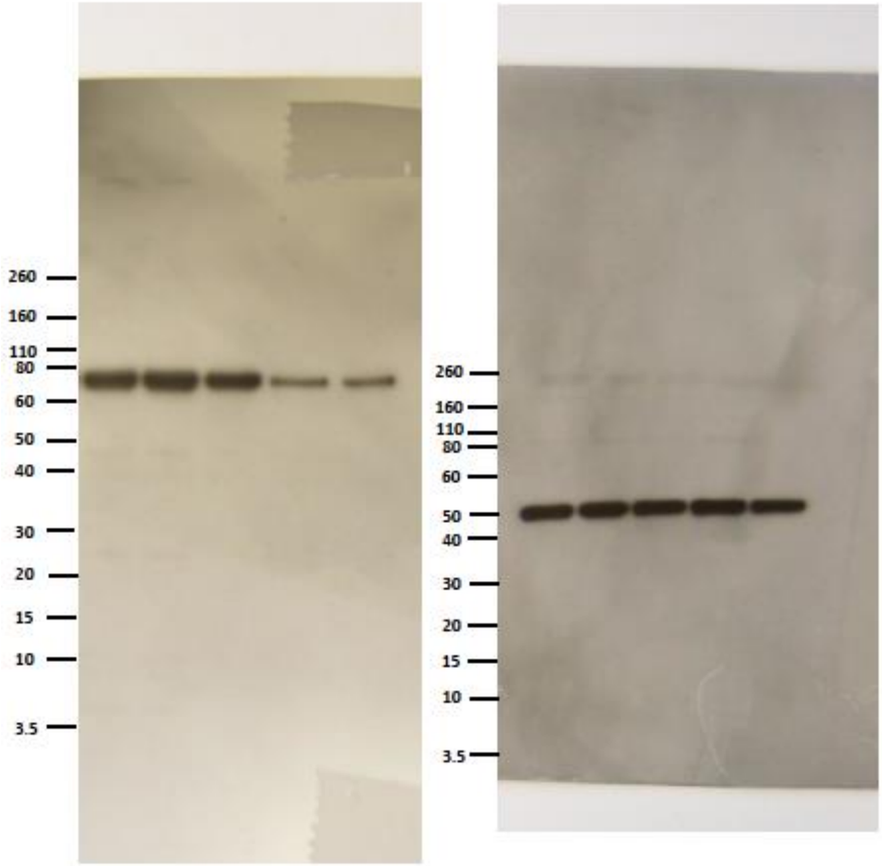

Original Western blots for Fig. 5c. Immunoblotted with rabbit monoclonal anti-BACE1 antibody (1:1000 dilution) (left panel) and detected with HRP-Goat-anti-rabbit IgG (H+L) secondary antibody (1:3000). Tubulin (right) was immunoblotted with anti-mouse-β-tubulin antibodies (1:200 dilution) prior to detection with the HRP-labelled antibody (1:3000 dilution). Lanes (from left to right): FAM-CGY+72 nM siControl, 72 nM siBACE1, FAM-CGY SP+72 nM siBACE1, FAM-CGY+48 nMsiBACE1 and FAM-CGY+72 nM siBACE1.

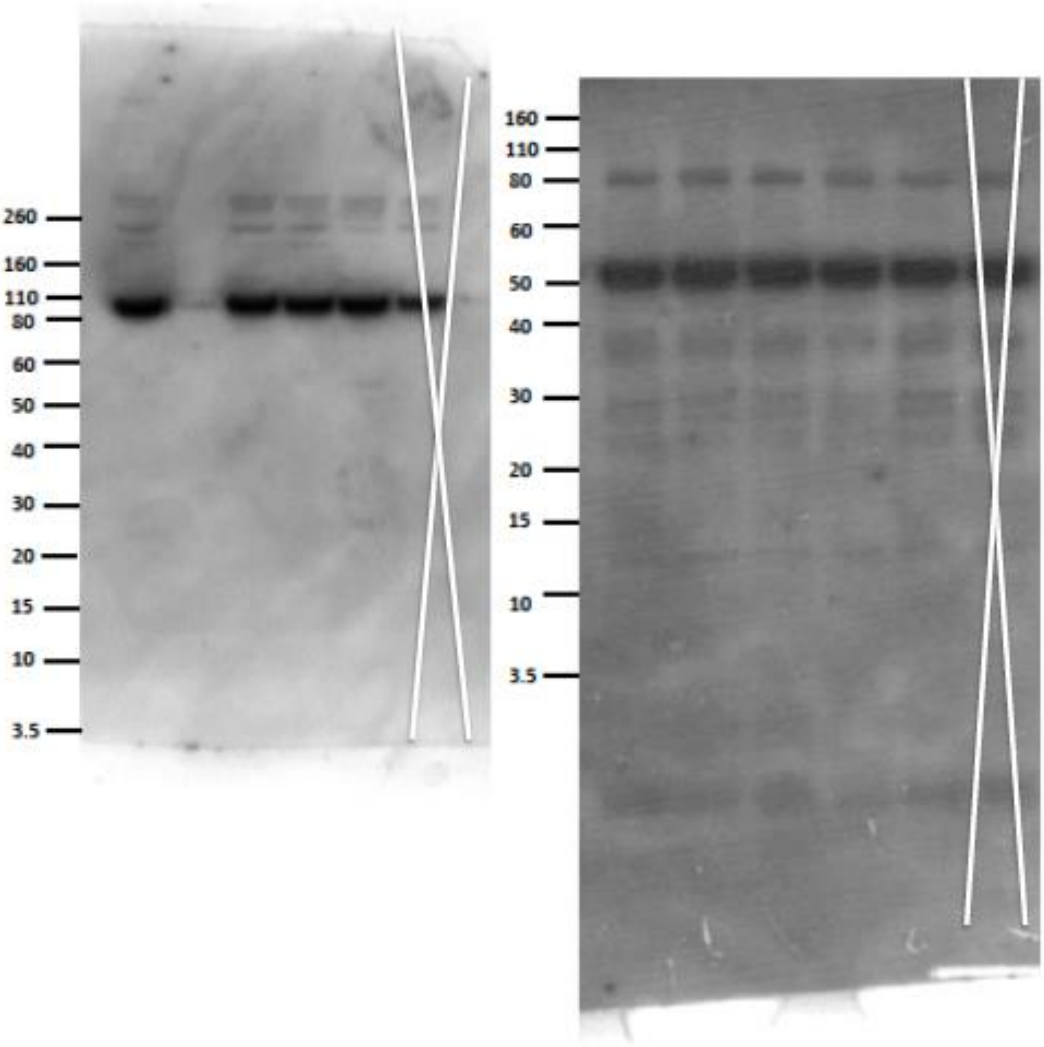

Original Western blot gels for Supplementary Fig. 16. Immunoblotted with mouse anti-human TfR antibody (1:200 dilution) (left panel) and mouse anti-human-β-tubulin (1:200 dilution) (right panel) and detected with HRP-Goat-anti-mouse IgG (H+L) secondary antibody (1:3000). Lanes (left to right): hCMEC/D3 cells, MCF-10A cells, MCF-7 cells, HeLa cells, HUVECs.
