# Supplementary material for: Crossing the blood-brain-barrier with nanoligand drug carriers self-assembled from a phage display peptide
Source: Nat Commun. 2019 Oct 11;10:4635. doi: 10.1038/s41467-019-12554-2 (PMC6789111; doi:10.1038/s41467-019-12554-2)
Supplement: Supplementary file 2 — Reporting Summary [file 41467_2019_12554_MOESM2_ESM.pdf]

nature research

Corresponding author(s): SM Moghimi; NCOMMS-18-28909A-Z

Last updated by author(s): 13 Sept, 2019

Reporting Summary

Nature Research wishes to improve the reproducibility of the work that we publish. This form provides structure for consistency and transparency in reporting. For further information on Nature Research policies, see Authors & Referees and the Editorial Policy Checklist .

Statistics

For all statistical analyses, confirm that the following items are present in the figure legend, table legend, main text, or Methods section.

n/a	Confirmed	
☐	☒	The exact sample size (*n*) for each experimental group/condition, given as a discrete number and unit of measurement
☐	☒	A statement on whether measurements were taken from distinct samples or whether the same sample was measured repeatedly
☐	☒	The statistical test(s) used AND whether they are one- or two-sided<br>*Only common tests should be described solely by name; describe more complex techniques in the Methods section.*
☒	☐	A description of all covariates tested
☐	☒	A description of any assumptions or corrections, such as tests of normality and adjustment for multiple comparisons
☐	☒	A full description of the statistical parameters including central tendency (e.g. means) or other basic estimates (e.g. regression coefficient) AND variation (e.g. standard deviation) or associated estimates of uncertainty (e.g. confidence intervals)
☐	☒	For null hypothesis testing, the test statistic (e.g. *F*, *t*, *r*) with confidence intervals, effect sizes, degrees of freedom and *P* value noted<br>*Give P values as exact values whenever suitable.*
☒	☐	For Bayesian analysis, information on the choice of priors and Markov chain Monte Carlo settings
☒	☐	For hierarchical and complex designs, identification of the appropriate level for tests and full reporting of outcomes
☒	☐	Estimates of effect sizes (e.g. Cohen's *d*, Pearson's *r*), indicating how they were calculated

Our web collection on statistics for biologists contains articles on many of the points above.

Software and code

Policy information about availability of computer code

Data collection	No software was used
Data analysis	Atomic force microscopy: Nanoparticle dimensions were determined using Image J software (http://rsb.info.nih.gov/ij/download.html) and reported as the mean size of at least 100 randomly selected images ± s.d.<br>Wild-field microscopy: Diffraction PSF 3D was used to calculate the point spread function followed by 3D deconvolution by Leica LAS AF Lite software.<br>Western blot analysis of receptor expression: The Bands were quantified with ImageJ 1.44p (http://imagej.nih.gov/ij/).

For manuscripts utilizing custom algorithms or software that are central to the research but not yet described in published literature, software must be made available to editors/reviewers. We strongly encourage code deposition in a community repository (e.g. GitHub). See the Nature Research guidelines for submitting code & software for further information.

Data

Policy information about availability of data

All manuscripts must include a data availability statement. This statement should provide the following information, where applicable:

- Accession codes, unique identifiers, or web links for publicly available datasets
- A list of figures that have associated raw data
- A description of any restrictions on data availability

There are no restriction on data availability. Supplementary file contain further supporting data, raw data on peptide synthesis and characterisation and uncropped SDS-PAGE gel images. Data Source file is appended. Accession codes and unique identifiers are not applicable to this work.

Field-specific reporting

Please select the one below that is the best fit for your research. If you are not sure, read the appropriate sections before making your selection.

- [x] Life sciences
- [ ] Behavioural & social sciences
- [ ] Ecological, evolutionary & environmental sciences

For a reference copy of the document with all sections, see nature.com/documents/nr-reporting-summary-flat.pdf

Life sciences study design

All studies must disclose on these points even when the disclosure is negative.

Sample size

Peptide synthesis and characterisation: 3 separate preparation and characterisation (preparative HPLC and mass spect) in each case.
SDS-PAGE experiments were repeated three times with three independent preparations of peptides.
AFM, TEM, critical aggregation concentration, Naoparticle Tracking (NTA) and zeta potential studies were repeated three times with different preparations of FAM-CGY.
Cell incubation studies were done in triplicate and each experiment was repeated three times, otherwise stated for each experimental set.
Animal studies: each animal group consisted of 3 mice and each experiment was repeated 3 times.
Fluorescent and immunofluorescent microscopy experiments of brain sections: Each experiment was repeated at least 3 times with different sections (n = 9).

Data exclusions

No data exclusion has been applied.

Replication

Each experiment was replicated 3 times and with success and similar trend in each case. Appropriate statistical analysis is provided for each experiment. Clearly defined error bars are presented and where appropriate sd or sem.

Randomization

Not relevant to this study.

Blinding

The researchers were not blinded due to complexity and large number of positive and negative controls and the nature of the experiments.

Reporting for specific materials, systems and methods

We require information from authors about some types of materials, experimental systems and methods used in many studies. Here, indicate whether each material, system or method listed is relevant to your study. If you are not sure if a list item applies to your research, read the appropriate section before selecting a response.

Materials & experimental systems

n/a	Involved in the study	
☐	☒	Antibodies
☐	☒	Eukaryotic cell lines
☒	☐	Palaeontology
☐	☒	Animals and other organisms
☒	☐	Human research participants
☒	☐	Clinical data

Methods

n/a	Involved in the study	
☒	☐	ChIP-seq
☐	☒	Flow cytometry
☒	☐	MRI-based neuroimaging

Antibodies

Antibodies used

For all reagents catalogue numbers (?number) are indicated in the Methodology section. Mouse anti-human transferrin receptor antibody (#A-11130), mouse anti-human-β-tubulin (#32-2600), HRP-goat-anti-mouse IgG (H+L) (#62-6520), and HRP-goat-antirabbit IgG (H+L) (#65-6120) were purchased from Life Technologies (CA, USA). Rabbit polyclonal to mouse and human RAGE (#ab3611), rabbit anti-mouse and human TfR antibody (#ab84036), rabbit anti-human claudin-5 antibody (#ab131259), anti-Tuj1 antibody (#ab78078, Abcam, UK), anti-GFAP antibody (#ab10062), mouse monoclonal to CD31 (#ab24590, reacts with mouse and human), rabbit polyclonal to CD31 (#ab28364, reacts with mouse and human), rabbit polyclonal to β-catenin (#ab16051, reacts with human), anti-BACE1 antibody (#ab108394), donkey anti-mouse IgG H&L (Alexa Fluor®680) (#ab175774), donkey anti-mouse IgG H&L (Alexa Fluor®450) (#ab175658) and donkey anti-mouse IgG H&L AlexaFluor555 (#ab150110) were purchased from Abcam (Cambridge, UK). Anti-Iba1 antibody ((#ab019-19741) was from Wako, Japan. Goat anti-rabbit IgG (H+L) Superclonal secondary antibody Alexa Fluor 555 (#A27039) was from ThermoFisher Scientific UK.

Validation

Antibodies verified by suppliers. Validation strategies are fully indicated and described in control experiments throughout the manuscript and supplementary file and data presented.

Eukaryotic cell lines

Policy information about cell lines

Cell line source(s)	The human brain endothelial cell line (hCMEC/D3) was obtained under licence from University Paris 05, CNRS, Institute Cochin, INSERM (Paris, France). Human breast cancer cell line (MCF-7) (ATCC® HTB-22™) and human mammary epithelial cell line (MCF-10A) (ATCC® CRL-10317™) were purchased from American Type Culture Collection (VA, USA).
Authentication	The hCMEC/D3 cell line was maintained and characterized in accordance to regularly updated protocols by Institute Cochin and certified mycoplasma free.<br>Al other cell lines were from American Type Culture Collection was accompanied by certificates and verified by the supplier.
Mycoplasma contamination	All cell lines were routinely tested for mycoplasma contamination and verified free of any mycoplasma contamination in all experiments.
Commonly misidentified lines (See ICLAC register)	No misidentified lines.

Animals and other organisms

Policy information about studies involving animals; ARRIVE guidelines recommended for reporting animal research

Laboratory animals	Male and females (50:50 distribution) ICR mice (6–8 weeks old, body weight 20–26 g). Animal protocols were in accordance with the "Animal Care and Use Committee Guidelines of Guangzhou Institute of Biomedicine and Health".
Wild animals	This study did not involve wild animals.
Field-collected samples	Free access to food and water; each group (n = 3) housed in separate cages; animals kept at controlled humidity and temp (room temp); controlled lighting (12 h light/dark cycles). Details of animal sacrifice is presented in the Methodology section.
Ethics oversight	Animal Care and Use Committee Guidelines of Guangzhou Institute of Biomedicine and Health, China.

Note that full information on the approval of the study protocol must also be provided in the manuscript.

Flow Cytometry

Plots

Confirm that:

- [ ] The axis labels state the marker and fluorochrome used (e.g. CD4-FITC).
- [ ] The axis scales are clearly visible. Include numbers along axes only for bottom left plot of group (a 'group' is an analysis of identical markers).
- [ ] All plots are contour plots with outliers or pseudocolor plots.
- [x] A numerical value for number of cells or percentage (with statistics) is provided.

Methodology

Sample preparation	FACS analysis: Our procedures only applies to the median cell fluorescence intensity and obtained by analysing 10,000 cells in a typical run and each run repeated at least three times.
Instrument	BD FACS Array™ Cell Analysis
Software	BD FACS Array™ Cell Analysis accompanied software.
Cell population abundance	No cell sorting. Not applicable to our experimental design.
Gating strategy	No cell sorting. Not applicable to our experimental design.

- [ ] Tick this box to confirm that a figure exemplifying the gating strategy is provided in the Supplementary Information.
